# Heavy Metal-Induced Cerebral Small Vessel Disease: Insights into Molecular Mechanisms and Possible Reversal Strategies

**DOI:** 10.3390/ijms21113862

**Published:** 2020-05-29

**Authors:** Jayant Patwa, Swaran Jeet Singh Flora

**Affiliations:** Department of Pharmacology and Toxicology, National Institute of Pharmaceutical Education and Research Raebareli (NIPER-R), Near CRPF Base Camp, Post Office Mati, Sarojini Nagar, Lucknow (U.P.) 226002, India; jayantpatwa93@gmail.com

**Keywords:** heavy metals, small vessel diseases, oxidative stress, endothelial dysfunction, ROS

## Abstract

Heavy metals are considered a continuous threat to humanity, as they cannot be eradicated. Prolonged exposure to heavy metals/metalloids in humans has been associated with several health risks, including neurodegeneration, vascular dysfunction, metabolic disorders, cancer, etc. Small blood vessels are highly vulnerable to heavy metals as they are directly exposed to the blood circulatory system, which has comparatively higher concentration of heavy metals than other organs. Cerebral small vessel disease (CSVD) is an umbrella term used to describe various pathological processes that affect the cerebral small blood vessels and is accepted as a primary contributor in associated disorders, such as dementia, cognitive disabilities, mood disorder, and ischemic, as well as a hemorrhagic stroke. In this review, we discuss the possible implication of heavy metals/metalloid exposure in CSVD and its associated disorders based on in-vitro, preclinical, and clinical evidences. We briefly discuss the CSVD, prevalence, epidemiology, and risk factors for development such as genetic, traditional, and environmental factors. Toxic effects of specific heavy metal/metalloid intoxication (As, Cd, Pb, Hg, and Cu) in the small vessel associated endothelium and vascular dysfunction too have been reviewed. An attempt has been made to highlight the possible molecular mechanism involved in the pathophysiology, such as oxidative stress, inflammatory pathway, matrix metalloproteinases (MMPs) expression, and amyloid angiopathy in the CSVD and related disorders. Finally, we discussed the role of cellular antioxidant defense enzymes to neutralize the toxic effect, and also highlighted the potential reversal strategies to combat heavy metal-induced vascular changes. In conclusion, heavy metals in small vessels are strongly associated with the development as well as the progression of CSVD. Chelation therapy may be an effective strategy to reduce the toxic metal load and the associated complications.

## 1. Introduction

Life expectancy is currently higher than at any other time in history, and is anticipated to rise persistently in industrialized countries. Consequently, age-associated morbidities will progressively pose serious challenges to the civilians and health care systems. Clinical evidences suggest that Cerebral Small Vessel Disease (CSVD) is a major contributor in 45% of dementia cases and approximately 20% in all strokes globally, which is a serious concern today due to the lack of an appropriate therapy [1,2]. The cost burden of CSVD is formidable to the society as the reasons for the illness are obscure, prevention, and effective treatments are not as good as expected [3]. Earlier, CSVD was considered innocuous, but has emerged as a notorious contributor in the vascular type of dementia, depression, cognitive dysfunction, and gait problems commonly observed in the patients challenged with CSVD [4]. There are several factors, including genetic and traditional, as well as environmental, such as pollution, pesticides, and heavy metals, which are considered as the major contributors to the development of CSVD [5,6]. Rich literature is available that suggests that heavy metal exposure is closely linked with vascular disease. However, the roles of environmental factors are poorly explored in CSVD. From a toxicological point of view, understanding small vessel tolerance against heavy metal-induced stress could reveal insight into the reasons for CSVD and other associated diseases. Heavy metals including cadmium (Cd), mercury (Hg), copper (Cu), and lead (Pb), and metalloid like arsenic (As) are well known environmental pollutants. Heavy metals, including Pb, Cu, Cd, and metalloid like (As)are considered as serious threat for human health due to their higher density and accumulation in the biological systems [7,8]. Abundant scientific reports indicate heavy metal pollution as a serious global health problem even at a lower concentration and in particular industrial developing countries. Humans get predominantly exposed to heavy metals via contaminated food, water consumption, or through inhalational. Water resources and air, on the other hand, get contaminated through industrial or agricultural waste, which generally contain high heavy metals concentration [9,10]. We thus hypothesized that exposure of Pb, As, Cu, Hg, and Cd could be among the major contributors to the development of CSVD and other related complications. There are several mechanisms involved in the heavy metal-induced CSVD; however, oxidative stress is considered as one of the leading mechanisms. Several studies in the past have reported that heavy metals significantly bind with the cytoplasmic, DNA, and nuclear proteins, leading to oxidative injury and damage to the biological macromolecules [11]. It has been well established that heavy metals, such as Cu, As, Cd, Hg, and Pb are capable of generating reactive radicals, like copper, is known to generate reactive oxygen species (ROS) through Fenton like reaction [12]. The reactive oxygen species (ROS) are formed due to the partial reduction of molecular oxygen. ROS, such as superoxide anion (O_2_−), hydroxyl radical (OH^•^), hydrogen peroxide (H_2_O_2_), and singlet oxygen (^1^O_2_), are produced as a product of the respiratory chain in mitochondria, in photochemical and enzymatic reactions, as a result of the exposure to heavy metal [13]. Past studies have demonstrated the possible role of inflammation in the heavy metal-induced vascular inflammation and explained that excessive ROS generation might activate the inflammatory pathway and, consequently, expedite the progression of CSVD [7,14].

In this review, we have discussed the impact of few selected heavy metals in the development and progression of CSVD, associated mechanistic pathways, and possible therapeutic strategies. We have also tried to elaborate how metals/metalloid induced oxidative stress affect CSVD, possible way of their biochemical evaluations, which might be helpful in planning a strategy to combat the toxic effects of metals by reducing unsafe heavy metal loads from the cell and, thus, achieve physiological recoveries in CSVD. As the list of heavy metals is long, we restricted this review to few major environmental contaminants/pollutants, like Cd, As, Pb, Cu, and Hg in the development of CSVD.

## 2. Cerebral Small Vessel Disease

CSVD is a broad term used to describe pathological complications associated with the small blood vessels including arterioles, capillaries, and small veins in the brain. Since most of the brain tissues look white on magnetic resonance imaging (MRIs) and, thus, in the past, these pathological conditions were alluded to as “white matter changes.” CSVD is most frequently observed image-related neurological complications, and plays a significant role in the etiology of at least three major diseases, vascular dementia, stroke, and gait decline [15]. Neuroimaging highlights CSVD incorporate blood–brain barrier (BBB) impairment, white matter hyperintensities, chronic inflammatory responses, recent small sub-cortical infarcts, perivascular spaces, lacunas, micro-bleeds, leukocyte infiltration, and brain atrophy are the classical pathological features of CSVD. The major clinical appearances of CSVD include stroke, cognitive impairment, mental retardation, psychotic disorders, abnormal gait, and urinary intemperance [16]. Despite several advancements in the neuroimaging field in the recent decades and also the development of novel biomarkers, the etiology of the pathogenesis of CSVD has still not been elucidated fully [4]. The inner wall of the blood vessels, generally covered with the monolayer cells of endothelium that create a functional and structural barrier among the circulating blood and the vessel wall, also play a critical role in the smooth functioning of the vascular system, and in cases of any impairments, cause vascular disorders [17]. Based on clinical studies, researchers also provided a link between damaged BBB with an early sign of the development of the CSVD in humans. In a few recent studies, the monogenic form of the SVD, particularly the cerebral autosomal dominant arteriopathy with sub-cortical infarcts and leukoencephalopathy (CADASIL), and ‘sporadic’ SVD, have been emphasized, which provided to some extent the molecular insight of the disease progression. On the basis of proteomic and biochemical examination of post-mortem monogenic CSVD patients, and also in animal models, it was concluded that extracellular matrix (ECM) dysfunction is one of the leading mechanisms. Further, the pathogenesis of CSVD is likely to begin with an expansion in the penetrability of the BBB with the development of Virchow Robin spaces (perivascular spaces), symptomatic lacunar infarcts, white issue injuries, and micro-bleeds as sequelae [18,19,20]. Astrocytes, pericytes, and endothelial cells play a major role in the maintenance of the BBB. Endothelial dysfunction is a primary cause of the increased BBB permeability [21]. This could be attributed to the fact that vascular endothelium regulates the passage of macromolecules and circulating cells from blood to tissues and, thus, become shighly vulnerable to oxidant stress, which is considered a key risk factor in the pathogenesis of various vascular diseases [22]. It is reported that nitric oxide critically regulates the vascular tone, but increases ROS load in response to several stimuli, including hypertension and hyperglycemia. Reactive oxygen species (ROS) are highly reactive chemical forms of oxygen, such as superoxide anion and hydroxyl radical. Several enzymes (oxidases) in the body are capable of forming superoxide anion (⋅O2−) from molecular oxygen. NAD(P)H oxidase, cyclooxygenase (COX), xanthine oxidase (XO), and nitric oxide synthase (NOS) are capable of forming superoxide anion from molecular oxygen. Increased oxidative metabolism, the absence of certain oxidase cofactors (e.g., tetrahydrobiopterin required by NOS), and inflammatory and disease conditions can lead to increased superoxide production. Superoxide radicals, which get converted into peroxynitrite resulting in the decreased bioavailability of NO and promoting endothelial dysfunction [23,24]. Vascular oxidative stress facilitates systemic inflammatory response in the brain through the immune activation. Activated immune cells travel into the vasculature and discharge immune signaling molecules like ROS, chemokines, cytokines, and matrix metalloproteinases (MMPs), causing damage to the vascular system and eventually promote vasoconstriction and remodeling of blood vessels [25,26]. Several studies reported that overexpression of MMPs also implicated in the dysfunction and remodeling of small blood vessels in the brain [27,28]. Further, it has been reported that oxidative stress is the leading cause of overexpression of MMPs [29]. It has been reported that overexpression of MMPs, specifically MMP-9, plays an important role in the pathogenesis as well as in propagation of CSVD [30]. It was noted clinically that the expression of MMPs was higher in the **Cerebrospinal fluid** (CSF) of CSVD and stroke patients. Similar findings were also observed in the animal model.

## 3. Risk Factors

### 3.1. Genetic Factors

Epidemiological studies have suggested several potential vascular risk factors, including genetic, traditional, and environmental that could contribute to the prevalence, development, and accumulation of the CSVD [5]. Genomic investigations revealed the significant impact of genetic factors and are also helpful in understanding the pathogenesis of CSVD [1]. The cerebrovascular ailment of all causes is an extremely heritable feature. In the past, genetics studies were conducted to evaluate the involvement of the gene in the CSVD and several genes have been identified including NOTCH3, HTRA1, CTSA, CSF1R, COL4A, COL4A2, GLA, which are related with the CSVD associated disorders [31]. An association between family history of stroke and chances of stroke recurrence in the offspring have been reported to be approximately three-fold while, major appearance of CSVD, SVD stroke and white matter lesions are 16% and 50% heritable, respectively [32]. Hereditary examination of both uncommon familial CSVD disorders and stunning sporadic late-life disease is a promising way to deal with comprehension and finding novel medicines for CSVD.

### 3.2. Traditional Factors (Co-Morbid)

**Figure 1:**
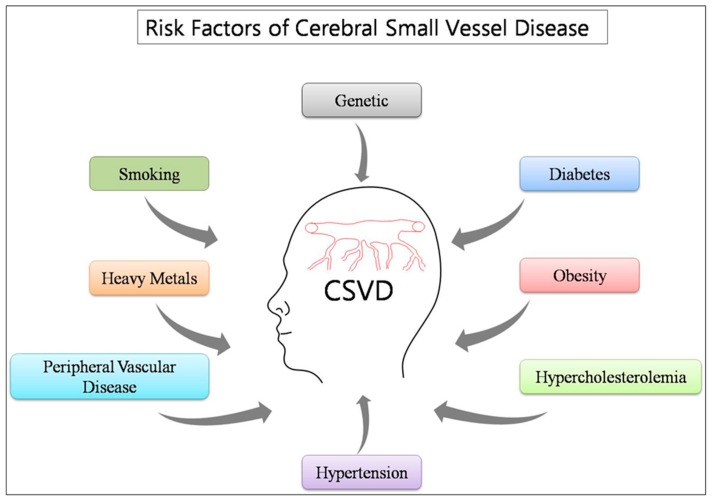
Schematic diagram of risk factors interacts to influence the development and progression of cerebral small vessel disease (CSVD).

Several reports have suggested that the most common traditional risk factors include age, sex, hypertension, diabetes, obesity, hypercholesterolemia, smoking, myocardial infarction, and peripheral vascular disease [33] (Figure 1). In few recently reported studies, hypertension emerges as the main risk factor among the other traditional factors [34]. It has been suggested that Alzheimer’s and vascular dementia share hypertension as the common factor between them [35]. In line with this, a cohort analysis of 463 people suggested hypertension and CSVD area dangerous mishmash, placing patients at increased risk for cognitive decline.

### 3.3. Environmental Factors

Researchers have correlated CSVD and its associated disorders with either genetics factors or traditional risk factors, but some other factors might also be significantly contributing to the ailments in humans that do not have such background. Numerous environmental risk factors have also been associated with CSVD associated disorders [36] (Figure 1). However, they are not directly causing the illness, but considered as the major contributor. According to the reports, various environmental risk factors have been suggested, such as air pollutants, heavy metals, pesticides, etc. [37,38]. There have been reports that mention the possible toxic effects of heavy metals, such as As, Cd, Pb, Hg, and Cu in CSVD associated disorders such as stroke, ischemia, dementia, amyloid angiopathy, etc. [39,40,41].

## 4. Neuroimaging Characteristics of Small Vessels Disease (SVD)

SVD changes the microstructure of the vascular system within the brain. The clinical features for SVD are not well established and its diagnosis is very difficult. However, the advancement of molecular brain imaging modality provides some opportunity to understand the progression of SVD. Ischemic strokes, hemorrhage episodes, and brain atrophy can be detected through brain imaging such as computed tomography (CT) in most of the patients. Due to slow progressing in nature, changes in the microvasculature within the different brain regions are usually ignored, which resulted in the development of small infracted areas (<20 mm in diameter and round-shaped) in cortical and sub-cortical brain regions. These small infarcts are considered as results from acute severe ischemic insult due to damage/blockage of a single perforating artery. This acute lacunar infarct is recognized as a “recent small sub-cortical infarct” [42]. The infracted lesions can also be detected early by diffusion-weighted imaging (DWI). DWI is a method to evaluate the micro architecture of brain-based on the patterns of random Brownian’s motion of water molecules [43]. This is highly sensitive in the early detection of acute hypoxic injury in the brain [44]. Additionally, white matter tract architecture can be regenerated on the basis of the degree of the spatial distribution of anisotropic diffusion using DWI. In DWI, the white matter lesions appear as hyperintense [45]. The recent small-infracted regions appear hypo-intense on the apparent diffusion coefficient map and either normal or hyperintense compared to the normal brain on fluid-attenuated inversion recovery (FLAIR)/T2 imaging in compassion to CSF [46]. Techniques, such as CT and magnetic resonance imaging (MRI), have limitations in the detection of an acute small ischemic lesion in 50% of patients. However, DWI is considerably sensitive to detect small-infracted regions within the first few hours and morphological changes, including infarct volume, diameters, and atrophy or finally cavity formation in the first 90 days of onset [47]. Leukoaraiosis, micro architectural characteristics of SVD and usually referred to as white matter hyper intensity (WMH), appears as hyperintense on a T_2_-weighted sequence on MRI and CT. WMH may also appear as isointense/hypointense on T1-weighted sequences, which is dependent on the severity of pathological changes [48]. Disturbances in small blood vessels also have a similar expression in imaging as BBB disruption and white matter, which are highly sensitive to hypoxic damage [49]. Older individuals show age-dependent vascular abnormality as denoted symmetrical hyper-intensities bilaterally on T_2_-weighted MRI [2]. Wardlaw et al have provided the standards for reporting vascular changes on neuroimaging (STRIVE). As per STRIVE recommendations, sub-cortical grey matter structures, such as the basal ganglia, may appear as hyper-intense and sometimes been analyzed alongside WMH [45,50]. WMH form vascular origin appears as hyper-intense on T_2_-weighted image and without cavitations on FLAIR-T2 imaging [49]. Lacuna and atrophy can be differed from general WMH using diffusion tensor MRI (DT-MRI) and magnetization transfer MRI (MT-MRI), which provides quantitative information of brain white matter. Kim et al have studied the relation with the amyloid load with the white network integration in SVD using MRI and DTI modality. Higher WMH volumes or lacunae numbers were found to be inversely correlated with white matter network integrations (higher numbers of short path length and reduction in global efficiency). White matter network segregation (higher values of clustering coefficient, transitivity, and modularity) is also proportionately evident with higher WMH and decreased cognitive performance [51].

Cerebral micro bleeds (CMBs) are also common in small vessel disease [19]. Micro bleeds are visible small (generally 2–5 mm in diameter) area of signal void on T_2_-weighted gradient-echo MRI, resulting from paramagnetic properties of focal deposits of hemosiderin-containing macrophages [52,53]. These perivascular hemosiderin masses are foci of the earlier hemorrhagic event resulted from macro-/micro-vessels involved in arteriolosclerosis [45,54]. Small hypo-intense lesions appear on paramagnetic-sensitive MR sequences with a ‘blooming effect’ (larger or more conspicuous on GRE than on spin-echo MRI). These lesions are generally not appears on FLAIR and CT. Round or oval-shaped micro bleeds lesions are most commonly seen in the cerebral hemisphere and cortico-subcortical junction. According to location, hemosiderin deposits are further categorized into three types: lobar, deep, and subtentorial CMBs [55]. Activation of MMPs is also evident in microbleed and cerebral hemorrhage. Myeloperoxidase was found to be directly correlated with MMP-9 activation in mice model of autoimmune encephalomyelitis. MRI is a useful modality in detecting MPO using MPO-Gd (MPO-specific molecular imaging agent). MMP-9 can be detected by fluorescence molecular tomography with MMP sense [56]. This approach can be used to detect the severity of SVD and the effect of a pharmacological intervention targeting MPO and MMPS. Similarly, the severity of SVD after ischemic stroke can be assessed using by CT scan along with leukoaraiosis, the number of lacunas and brain atrophy as a combining feature [57]. Brain atrophy is an alternative and promising surrogate marker for SVD that may also be computed from conventional MR sequences. This indicates a decrease in brain volume, which is not related to known injuries, such as head trauma and cerebral ischemia. In general, atrophy manifest as a bilaterally symmetrical reduction in total brain (gray and white matter) volume with larger ventricle size and volume [58]. In the elderly, WMH frequently occurs together with atrophy and manifested along with dementia and neurodegeneration [59]. To summarize, diagnostic criteria for SVDs are not clearly defined. SVD is more prevalent and can now be better diagnosed with the development of new brain imaging tools. MRI remains the main imaging modality as it has higher sensitivity and specificity for detecting both pathological alterations and progression of the SVD. It permits to detect vascular lesions commonly attributed in sub-cortical areas as well as lacunas in deep brain areas. Leukoaraiosis can be detected as numerous punctuates or confluent lesions and micro bleeds in the white matter by MRI. However, both computed tomography and MRI are able to detect morphological changes, but the functional consequence of these changes remains a concern from imaging modalities. Sometimes additional pathological co-occurrence such as levy bodies and amyloid β leads to diagnostic confusion in MRI. Positron Emission Tomography (PET) modality may provide some additional benefits in the segregation of vascular and degenerative basis of a decrease in cognitive functionality.

## 5. Role of Heavy Metals in CSVD

### 5.1. Lead (Pb)

Lead (Pb) is regarded as one of important environmental toxicants and most widely used metal in manufacturing industries including batteries, paints and pigments, plastic, ceramic, secondary foundries, and welding materials in many countries; moreover, Pb exposure is arguably the oldest acknowledged occupational health hazard [60,61]. Human exposure to Pb is mostly through contaminated food, water consumption, and air pollution aroused by industrial emission and gasoline containing lead compounds [62]. Pb intoxication severely affects various body systems predominantly CNS, hematopoietic, and renal systems [63]. The half-life of Pb is about several weeks in blood, whereas in the brain, it is nearly two years, while in bone it remains for decades [64]. CNS is the most susceptible to Pb toxicity and several neurological complications are associated with it, including vascular dementia, white matter disease, endothelial dysfunction, stroke, mood disorder, BBB impairment [38,65]. It is reported that Pb can easily cross the BBB through the Ca ATPase pump and even causes the abnormalities in the BBB permeability [66] (Table 1). Inconsistent with this in-vitro report, Pb affects the hCMEC/D3 endothelial cells and oxidative stress could be a major causative factor in endothelial dysfunction [67]. It is reported that Pb can bind with the thiol group of amino acid and protein including glutathione (GSH) resulting in dyshomeostasis of thiol-redox status and disturb the cellular physiology [68]. Chronic exposure to Pb may cause deficiency of antioxidants by lowering the expression of various cellular antioxidant defense enzymes, such as catalase, GPx, and superoxide dismutase (SOD). These changes can be attributed to the fact that Pb may disturb the antioxidants activity by replacing their active cofactor metals [69]. Lead exposure can also increase the cellular copper/iron level possibly due to their replacement from various metalloproteins, resulting in a higher cellular amount of free redox-active metals and, thus, augments the ROS generation through the Fenton-like reaction [12]. The precise mechanism by which Pb induced impairment in BBB permeability is obscure yet. There have been reports that highlight that occludin protein actively participates in the maintenance of tight junction of endothelial barriers [70,71]. Further, altered expression in this protein could cause the opening of inter-endothelial tight junctions. It has proven by Wang et al., who investigated the occludin expression on lead exposure in the rat brain, and found that occludin expression was significantly decreased in Pb intoxicated animals [72]. Additional evidence from a case study further suggested that childhood lead exposure is strongly linked with a region-specific reduction in adult gray matter volume confirmed from the MRI images analysis of participants [73]. Gray–white matter abnormalities are greatly linked with the small vessel disease [74].

### 5.2. Copper (Cu)

Copper is a well-known ecotoxicant and humans get exposed to copper via consumption of water, food, and by inhalation of industrials copper dust. Copper plumbing is a major source of higher copper levels in the water. The recent findings suggested that agricultural workers get exposed to Cu by handing Cu-based agrochemicals and Cu-IUDs is one of leading cause for higher Cu levels in the women [84,85]. Cuions are the key structural component of around 30 enzymes that control several functions in eukaryotes. Transition property of Cu ion makes it an ideal enzymatic cofactor; however, this redox and coordination chemistry also proved deleterious to the cell [86,87]. Cu transporters including CTR1, ATP7A play a major role in the trafficking of Cu to CNS [88]. Recently, copper targeting approaches have been suggested for the life-threatening diseases like Cerebral amyloid angiopathy (CAA), CSVD, chronic lung inflammation and neurodegenerative disorders including Alzheimer’s disease (AD), Parkinson’s disease (PD), Huntington disease (HD) and Prion disease [89,90]. Elevated copper levels in CSF and different brain regions of AD patients provide direct evidence of its close association in the development and progression of AD [91]. Wilson disease is a genetic disorder associated with abnormal copper metabolism, resulting in increased copper deposition in the targeted organs, such as liver and brain [92]. MRI images of the Wilsons Disease (WD) patients have also revealed that copper caused white matter abnormalities [93], a hallmark of the CSVD [74]. A higher copper level is linked with the cerebral endothelial dysfunction, which can be correlated with the in-vivo studies, which investigated the role of copper nano-particles on rat cerebral micro-vessel endothelial cells (Table 2). The study reported that at the lower dose copper gets involved in the proliferation, whereas at the higher dose, induces blood–brain barrier toxicity and potential neurotoxicity [94]. Oxidative stress is one of the key mechanisms involved in copper associated disorders [95]. It has been observed that excess copper caused hypermethylation of amyloid-beta peptide and in the mild acidic condition starts to precipitate on the small vessel and caused cerebral amyloid angiopathy [96]. These metal and peptide precipitates are aggregated leading to ROS generation, especially hydroxyl radicals, and caused vascular dysfunction [97]. These findings can be correlated with a case study, which investigated the role of copper in AD and vascular dementia. The study concluded that copper level was increased in the AD patients and thus explained the possible Cu mediated amyloid-beta toxicity in the brain [98]. Interestingly, a case control study, which investigated serum copper level in Chinese stroke patients, concluded that an elevated copper is linked to the endothelial dysfunction, increased ROS generation, and the processes of inflammation, leading to the formation of carotid plagues and may also contribute to the cerebral ischemic injury [99].

### 5.3. Mercury (Hg)

Mercury (Hg) is another toxic metal, which is serious health hazard to the humans [109]. It exists in specifically three different forms, elemental, inorganic, and organic. Inorganic mercury occurs in the metallic (Hg^°^), mercurous (Hg^+^), and the mercuric form (Hg^++^). Aquatic microorganisms have the ability to convert both the cations in the numbers of organic forms through the methylation mechanism [110]. These organic forms of mercury bio-accumulate in the food chain and thus people who consume highly contaminated seafood, including fish, shellfish, getsseverely affected [111]. The brain is a highly susceptible organ to methyl-mercury and sufficient evidences are linked with several vascular disorders including damage to BBB, vascular dementia, endothelial dysfunction, white matter hyperintense, and stroke [112,113,114,115]. Earlier studies concluded that multifactorial mechanisms have been suggested in Hg induced toxicity and oxidative stress is one of primary mechanism involved in the BBB permeability impairment, which modifies Hg induced neurotoxicity [40,116]. In-vitro investigations have proven that MeHg exposure reduced the proliferation of endothelial cells [117,118] by decreasing the expression of fibroblast growth factor-2 [119]. The expression of vascular endothelial growth factor (VEGF) and VEGF receptor-1/-2 was upregulated in the endothelial cells following MeHg intoxication. VEGF, which plays a significant role in endothelial cell migration, proliferation, and maturation, induces hyperpermeability of vessels, eventually causing vascular leakage and edema [120]. In line with these finding Wiggers et al., reported that chronic exposure to HgCl_2_ in Wistar rats’ exhibited cerebral vasospasm and suggested the possible role of decreased NO bioavailability. The study also explained that the exposure to Hg increased the ROS load in the vessels through NADPH oxidase mediated pathway. They also reported that COX-derived prostanoids expressions reduces NO bioavailability, which might be responsible for the smooth functioning of blood vessels (Table 3) [121]. Rich scientific evidence is available that support the involvement of Hg in Alzheimer’s diseases and also explain several associated insight like molecular mechanism including oxidative stress, Neuroinflammation, cholinergic and serotonergic transmission, amyloid plague formation, selenium depletion and epigenetic changes [122,123]. The high Hg contents were found in the Alzheimer’s disease patients’ brain regions and also in blood [124].

### 5.4. Arsenic (As)

Arsenicisa ubiquitous metalloid and has long been known as toxic to humans. The main sources of chronic arsenic exposure to humans are through inhalation of metal dust and drinking water [131,132]. Its toxicity is associated with various clinical manifestations such as skin lesions, chronic lung disease (bronchitis, COPD, and bronchiectasis), liver disease, diabetes, edema of limbs, and congestion of eyes, polyneuropathy, erectile dysfunction, anemia, and vascular abnormalities and collectively known as arsenicosis [133]. Acute arsenic exposure is known to cause diabetes, various neurological symptoms such as tremor, hyperpyrexia, convulsion coma, etc. [134,135]. Hypertension, ischemic heart disease, and cerebrovascular abnormalities are important vascular anomalies associated with chronic arsenic toxicity. Arsenic exists, mainly in arsenite and arsenate forms, and both are toxic. Aposhian et al. reported the metabolism of inorganic arsenic through methylation into dimethylarsinous acid, which excreted through urine [136]. Trivalent ionic arsenic (As^3+^) is biotransformed into less toxic arsenicals (As^5+^) through methylation by arsenic methyltransferase, in the presence of S-adenosylmethionine [136].In line with an earlier report, Douillet et al. observed the association of arsenic specific methyltransferase (AS3MT) with the development of arsenic-induced metabolic alterations in mice. AS3MT knockout mice gained higher weight and developed insulin resistance compared to wild-type controls on arsenic exposure. These evidences support that the methylation and GSH mediated reduction, detoxify the toxic arsenic. Arsenic detoxification further depletes brain GSH [137]. Arsenic and its metabolite are accumulated in the kidney, lungs, heart, spleen, and brain of humans; and viral infection may further enhance the accumulation in the brain [138,139]. Wang et al. reported dose-response relationship in the development of carotid atherosclerosis on chronic exposure to inorganic arsenic [140]. Studies have also shown up-regulation of amyloid precursor protein gene transcription and tau phosphorylation and increased the rate of neuronal necrosis and apoptosis. Exposure to arsenic at environmentally relevant concentrations also caused ultra-structural changes in the brain [141,142,143].

Vascular dementia (vascular hypothesis of Alzheimer’s disease) is the result of reduced cerebral perfusion and impair neuronal functions. It is associated with various risk factors, such as cerebrovascular atherosclerosis, hypertension, diabetes, obesity, etc. The cerebral pre-ischemic and ischemic conditions have been reported to aggravate the production of amyloid precursor protein and amyloid β. Restricted perfusion further worsened the condition by impairing the clearance of this protein. There are several lines of evidence, which indicate the association of cardiovascular disease and arsenic exposure. Increased mortality rates have been reported on exposure to arsenic in the condition of circulatory disease (cerebrovascular disease, ischemic vascular disease, hypertension, etc.) [144]. Wu et al. reported that the increased mortality on arsenic exposure is the combined effect of all vascular diseases together (cardiovascular, cerebrovascular, disease of arteries and capillaries) [145]. A high level of monomethylarsonic acid has been reported with a significantly high occurrence of ischemic stroke incidence [146]. The Strong Heart Study, and other cohort studies, have demonstrated that low-to-moderate exposure to inorganic arsenic is positively associated with high occurrence of plaques and an increase in the thickness of carotid intima-media [147,148]. In these studies, higher levels of arsenic metabolite monomethylarsonic acid have been observed with increased carotid intima-media thickness. Endothelial dysfunction is a hallmark for various vascular disease including cerebral stroke and ischemia [149]. Furthermore, endothelial dysfunction alters blood–brain barrier physiology, reduced arterial perfusion, and change in the WMH sensitivity are considered as pivotal mechanisms of brain small vessel disease [150,151,152,153]. Zarazúa et al reported the effects of the detrimental effect of arsenic exposure on NO (nitric oxide) production, which is a critical modulator for endothelial function [154]. Arsenic-containing lipids also enhanced the permeability of the blood–brain barrier and may alter the arteriolar perfusion, hallmark characteristics of the small vessel disease [155,156]. Arsenic induced decrease in NO production was accompanied by significantly higher levels of lipid peroxidation and ROS production. These evidences provided the etiologic significance of arsenic in the development of small vessel disease of the brain through endothelial dysfunctions.

In addition to the micro structural changes in the brain, arsenic exposure also affects neuronal system at the cellular levels. Arsenic significantly affects the neuronal, glial, and astrocyte components of the brain [157,158]. The neurotoxic effects of arsenic are mediated through the oxidative stress and mitochondria dysfunction [152,159] (Table 4). It inhibits the mitochondrial complexes I, II, and IV of the electron transport chain, which increases ROS. As microglia is sensitive to arsenic toxicity [As (III)], the mitochondrial disturbance may induce apoptosis in microglial cells [160,161]. Arsenic has shown to induce alterations in the arachidonic acid metabolism and induces neuronal damage and inflammatory response in mice [162]. Monomethylarsonous acid at the sub-toxic doses also up-regulates the inflammatory cytokines gene in astrocytes [163]. There are convincing evidences available, which indicates the involvement of vascular inflammation in the development of cerebral microbleed, particularly in the stroke patients (CMB). It is further noteworthy that the hallmark characteristics of SVD (i.e., WMH, lacunas and atrophy) and are correlated with the inflammation and oxidative stress.

### 5.5. Cadmium (Cd)

Cadmium (Cd), a naturally occurring heavy metal is used in household electronics devices (television screens, lasers, etc.), batteries, and cosmetics. It was also used in paint pigments, welding and water pipes. It is a biologically non-essential metal and usually exists as a divalent salt such as CdCl_2_. Recently; Chen et al. have found that Cd metal accumulates at the high amount in liver testis, lungs and at a low amount in brain and serum for weeks on inhalation exposure to the mice similar to the study by Choudhuri et al. [172,173]. Similarly the accumulation of Cd has also reported in red squirrels inhabited the Cd-mines area with a higher level of brain oxidative stress [174]. Cellular oxidative damage is the major contributor to the toxicity induced by the Cd. Zheng et al had demonstrated the acute toxic effect of Cd on the zebrafish brain. In this study, 24 h exposure of Cd exhibited alteration of oxidative stress markers (Cu/Zn-SOD, CAT, and iNOS) and brain inflammatory markers (COX-2, NF-κB, Keep1, COX-2) at all transcription, translation, and post-translation levels [175]. In line with similar results, Cd had also reported altering brain (glial and neuronal cells) and mitochondrial morphology in zebrafish on incubation at a lower dose [176,177]. Cadmium induced oxidative stress may damage brain lipids and protein, but it’s interesting to observe that a well-known toxicant like chlorpyrifos has acted like an antagonistic to Cd [178]. Adefegha et al. have reported that oral administration of Cd for 21 days may increases brain cholinesterase (both AChE and BChE) along with Na^+^/K^+^-ATPase) and monoamine oxidase activity [179]. Cholinesterase and Na^+^/K^+^-ATPase have been demonstrated for their involvement in the neurodegenerative diseases and also have considerable pharmacological relevance [180,181]. As stated above, brain inflammation further favors the initiation and progression of different brain diseases including cerebrovascular diseases [182]. Phenolic like compound ferulic acid has been found to have a beneficial effect on Cd-induced increase in these brain enzymes and further provide a pharmacological relevance of oxidative stress and inflammation-mediated toxicity induced by Cd [179]. Additionally, Metallothionein (MT), a low molecular weight cysteine-rich protein that maintains levels of essential metal ions in the brain, was found to be downregulated on exposure to Cd directly and indirectly through oxidative stress [183]. Antioxidant like alpha-lipoic acid offers neuroprotection against oxidative stress-mediated Cd-toxicity through the upregulation of MT3 [183]. Cadmium exposure affects the microstructure of the brain along with general toxic effects like oxidative stress. Cd modifies neuronal morphology, the survival of neurons and consequently affecting cognitive functionality. The administration of Cd led to a decreased density of dendritic spines and an increase in caspase-3 and 9 immunoreactivities in hippocampal [184]. Yang et al. investigated the effect of Cd exposure on brain microstructure using juvenile mice. In this study, serious hyperemia of cerebral blood capillary in the piamater, microbleed of eosinophil, leukocyte, and increasing apoptotic cells were observed on Cd exposure for 10 days to juvenile mice. Cd also found to have altered the neuronal synapsis ultrastructure as evident from decreased synaptic cleft, fused presynaptic and postsynaptic membrane [185]. Earlier, López et al. have reported induction of both necrosis and apoptosis mediated neuronal death on Cd exposure [186]. The higher concentration of Cd can induce both depletion of intracellular ATP depletion and ATP release, however, at the lower doses, Cd caspase-3 mediated apoptosis in neuronal cells [186,187]. In an electron microscopy study, prenatal exposure to Cd also produced degenerative effects in the brain, such as cytomembrane disappearance and degeneration of organelles and vacuoles [188]. Moreover, the effects of Cd exposure on endothelial dysfunctions have been investigated by Ibiwoye and colleagues. Disrupted immune reactivity for endothelial barrier antigen (EBA) a marker of barrier-competent microvessels was observed on Cd exposure toasingle dose. Moreover, ill-defined astrocytic border, blurred white, and gray matter cytoplasm further indicated a disruption of endothelial function, blood–brain barrier, and microvessels [189]. Cd also induces mitophagy through the PINK1/Parkin pathway in brain [190]. In an autoradiographic investigation, cerebral vasculature found to be the primary target for Cd uptake during neonatal development [191] (Table 5). These evidences indicate that short term or long-term exposure to Cd may induce brain vascular pathology in relevance of small vessels disease.

## 6. Molecular Mechanisms

### 6.1. Oxidative Stress

**Figure 2:**
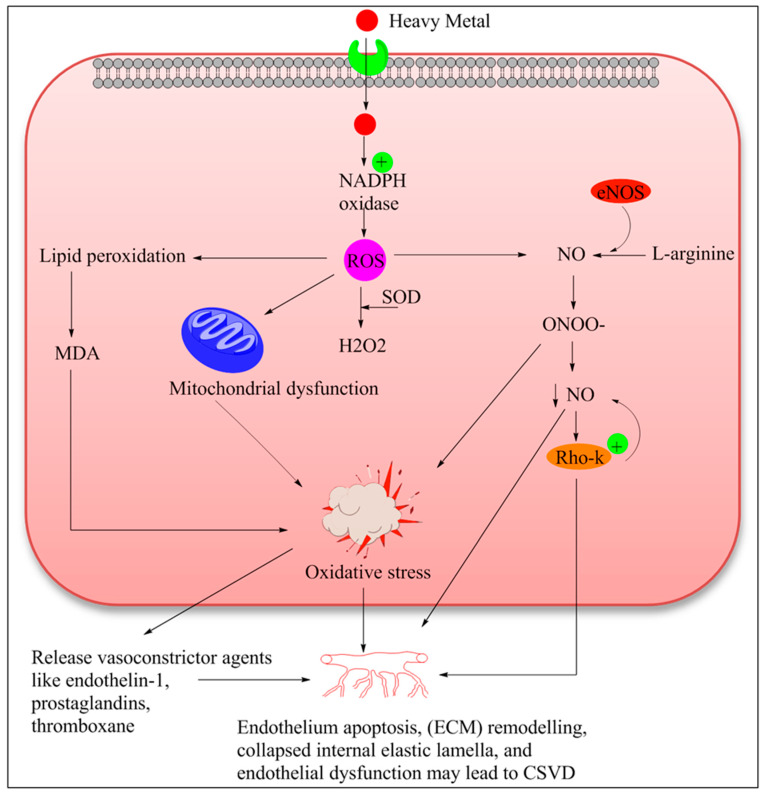
Mechanism of cerebral endothelial dysfunction imparted by heavy metal intoxication. Heavy metal can generate reactive oxygen species (ROS)by either direct (Fenton reaction) or indirect pathway (activating NADPH oxidase). Reactive oxygen species bind with the NO and rapidly convert into the peroxynitrite resulting in decreases in the bioavailability of NO in endothelial cells. Reduced NO level activates the Rho-kinase. Elevated ROS activity causes the lipid peroxidation of endothelial cells and thus produces the MDA, which is further toxic to the cell. On the other hand, ROS converts in H_2_O_2_ catalyzed by SOD. Finally, the reunion of all these events leads to oxidative insults to the endothelial cell and eventually causes endothelial dysfunction. Abbreviations: ROS: Reactive oxygen species, NADPH oxidase: nicotinamide adenine dinucleotide phosphate oxidase, SOD: Superoxide dismutase, H_2_O_2_: Hydrogen peroxide, MDA: Malondialdehyde, NO: Nitric oxide, ONOO: Peroxynitrite, Rho-k: Rho-associated protein kinase.

ROS is an umbrella term that covers several reactive oxygen species, namely superoxide, peroxynitrite, hydroxyl, and their metabolic species, basically derived from the molecular oxygen. Augmented level of different ROS elicits cellular and molecular damage and subsequent decrease level of antioxidant defense enzyme, denoted as oxidative stress [194]. In fact, oxidative stress is associated with a decreased life expectancy and numerous aging disorders (e.g., neurodegenerative disorders cardiovascular diseases, and metabolic diseases) [195]. Further, it is linked with the small vessel disease because a blood vessel is continuously exposed to the various stimuli [196]. The majority of researchers are in favor that oxidative stress is critically implicated in heavy metal-induced vascular complications [12,197]. Heavy metals are able to produce ROS directly through Fenton like reactions or indirectly such as increasing the free level of redox transition metal as well as stimulating the activity of NADPH oxidase, which catalyzes the ROS generation [198]. Following the elevated ROS load not only damages the cellular macromolecule, but, moreover, causes dyshomeostasis to key redox-dependent signaling processes in the small vessel wall. Perhaps the most widely elucidated mechanism by which oxidative stress can promote small vessel disease is through the interference of the vasoprotective nitric oxide (NO) signaling pathway [199]. Nitric oxide (NO) signaling pathway maintains the vascular tone, while during the ROS exposure superoxide species rapidly combines with NO and form peroxynitrite leading to the unavailability of NO eventually causing endothelial dysfunction [200]. It is reported that peroxynitrite is a much more powerful oxidant capable of damaging proteins and DNA and may further intensify the vascular dysfunction (Figure 2) [201]. Endothelial nitric oxide synthase (eNOS)/nitric oxide (NO) signaling pathway-dependent cerebral endothelial dysfunction is a major underlying mechanism to prelude vascular dementia which is associated with the CSVD [202]. Several in-vitro and in-vivo investigations have proved that exposure to heavy metal targets the nitric oxide (NO) signaling pathway, andinduces endothelium dysfunction [7]. An additional critically significant outcome of diminished NO bioavailability during disease impairment of RhoA kinase signaling. RhoA is important to EC migration, angiogenesis, and endothelial permeability [203]. Accumulating evidence indicates that elevated ROS level, decreases the availability of NO and increases peroxynitrite level, which might trigger RhoA kinase expression. FurtherRhoA kinase activation leads to the downregulation of eNOS expression and consequently decreases the level of NO leading to vascular dysfunction [204,205]. Heavy metal toxicity and RhoA kinase activation are still rudimentary, however, based on the literature, it could be speculated that heavy metal may induce ROS generation and ROS overload is directly linked to RhoA activation [206]. In fact, an increment in ROS activity increases the lipid peroxidation of the endothelial wall of small vessels, consequently participating in endothelial dysfunction. Malondialdehyde (MDA) is formed, which is an end product generated by decomposition of arachidonic acid, and also highly toxic to the cell. There have been reports that correlated increased MDA with endothelial [207] as well as vascular dysfunction in the middle-aged and elderly community-dwelling persons [208]. It is reported that in an activation of state endothelial cells has higher mitochondrial contents especially at the BBB. Furthermore, the heavy metal induces excessive ROS load that may lead to mitochondrial damage, which may cause the impairment of respiratory chain reaction and also in ATP synthesis. Thus, it is possible that, dueto energy deprivation, vascular endothelial cells may undergo apoptosis. An in-vitro study examined the role of heavy metal (Cd^2+^, Hg^2+^, and Cu^2+^) induced mitochondrial dysfunction depend cell death [209]. Furthermore, Tang et al.explained the molecular insight of Cd endothelial dysfunction in the human pluripotent stem cell and also highlighted EC apoptotic cell death pathway [210]. Responding to the oxidative stress event, endothelial cells may become activated and start to release vasoconstrictor agents like endothelin-1, prostaglandins, and thromboxane. These agents may be responsible for instigating inflammatory responses. Further activated endothelium cell significantly expressed the adhesion molecules and discharge chemokines like chemokine (C-C motif) ligand 2 (CCL2) to attract immune cells [211]. The increased level of chemokines and proteases in endothelial cells makes an interminable circle supporting the provocative response [212]. During chronic stress and vascular inflammation as well as variety of changes in endothelium, including apoptosis, extracellular matrix (ECM) remodeling, collapsed internal elastic lamella, and endothelial dysfunction may lead to small vessel associated complications [213,214]. Other possible mechanisms of heavy metal mediated vascular damage have been described during the inflammatory pathway, MMPs expression (Figure 2).

### 6.2. Inflammation

**Figure 3:**
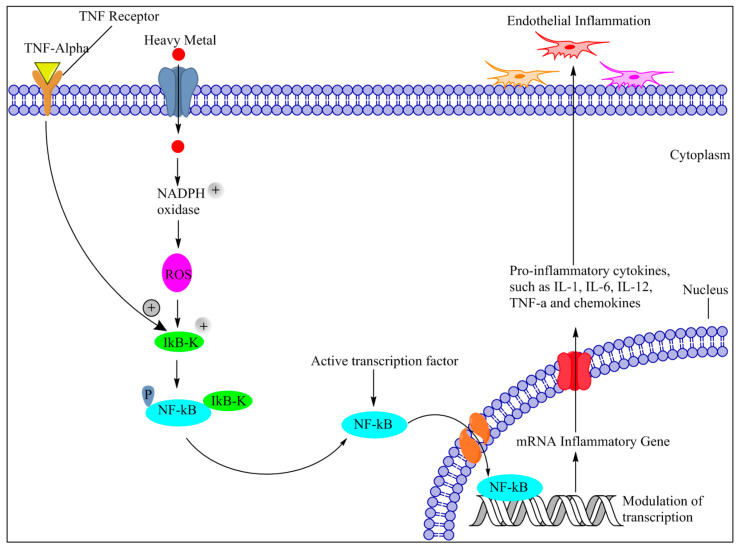
Mechanism of heavy metal imparts endothelial inflammation. ROS generation induced by metal exposure from various pathways, which activates an inflammatory cascade via NF-kB, mediated signaling, which triggers the inflammatory gene transcriptions and augments the expression of the pro-inflammatory cytokines such as (TNF-α, IL-1, IL-6, and IL-12). Further, TNF-activation phosphorylated IkB and induced activation of the NF-kB pathway. Thus aggravates the inflammatory and oxidative cycle leading eventually to endothelial dysfunction and promoting CSVD. Abbreviations: TNF: Tumor Necrosis Factor, IL-1: Interleukin-1, IL-6: Interleukin-6, IL-12: Interleukin-12, ROS: Reactive oxygen species, NADPH oxidase: nicotinamide adenine dinucleotide phosphate oxidase, IkB kinase: Inhibitor of kB, NF-κB: nuclear factor kappa B.

It has been mentioned that Reactive oxygen species (ROS), are produced in response to the heavy metals exposure. Considerable literature suggest that chronic oxidative insult to the small arteries leads to endothelial dysfunction and also elicits local as well as diffuse inflammation [214,215]. Nevertheless, inflammation may not be a negative phenomenon every time, as it also provides protection against toxic environments. Importantly if the pro-inflammatory compound rose while a decrease in the anti-inflammatory compound will create an imbalance that might establish an inflammatory state [216]. Emerging evidences reveal that heavy metal exposure imparts ROS mediated inflammation in the endothelial cell. Nuclear factor-kappa B (NF-κB) is a major transcription factor that regulates the pro-inflammatory gene and also plays a significant role in the production of pro-inflammatory cytokines such as tumor necrosis factor-α (TNF-α), IL-1β, IL-6, and IL-8. Increased ROS load instigates IκB kinase (IkK) pathway, resulting in a course of initiations, for example, MAP kinase, c-Jun amino-terminal kinases (JNK), and TNF receptor-related factor 1 (TRAF1) and 2 (TRAF2) [217] (Figure 3). As a result of these activations, NF-κB is trapped in the cytoplasm of the stimulated cell and subsequently translocated to the nucleus in response to the oxidative stimuli. In the nucleus, NF-κB triggers the target gene transcriptions as TNF-α, IL-1β, IL-6, and IL-8 (Figure 3), thus, induces vessel inflammation [218]. Outcome from a large number ofin-vitro and in-vivo investigations suggest that exposure to As and Pb, ROS activity in the epithelial cell of the vascular system further activates the downstream signaling molecule like IκB kinase (IkK). This could accelerate the translocation of NF-κB cytoplasm to nucleus where NF-κB nuclear translocation, it binds with the DNA and induces the transcription of pro-inflammatory cytokines [219,220,221]. The meta-analysis study concluded that exposure to As can activate NF-κB signaling pathway [222]. Interestingly, TNF-α activation can also elicit several inflammatory cytokines and chemokines expression through the activation of transcriptional factors, such as NF-κB and activator protein1 (AP-1) [218]. It is reported that TNF-activation principally appears to reduce the bioavailability of NO by (i) reducing the production of NO and, (ii) enhancing the removal of NO. It could be hypothesized that TNF-α-mediated crosstalk can trigger and expedites vascular inflammation, vascular remodeling, endothelium apoptosis vascular oxidative stress, and impaired NO bioavailability [223].

### 6.3. MMPs Expression

**Figure 4:**
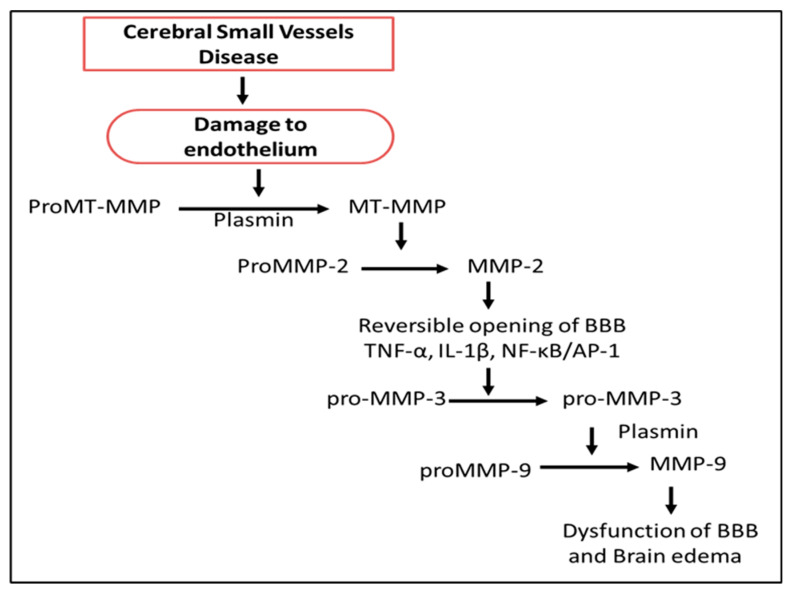
Role of matrix metalloproteinases (MMPs) in endothelial dysfunctions. Abbreviations: MT-MMPs: Membrane-type matrix metalloproteinases, TNF: Tumor necrosis factor, IL-1: Interleukin-1, NF-κB: nuclear factor kappa b, AP-1: Activator protein 1, BBB: Blood brain barrier.

Matrix metalloproteinases (MMPs) are the member of the Ca^2+^-Zn endopeptidase family with serve diverse role in the organism. MMPs have been sub-classified in six subfamilies, viz., collagenase, gelatinases, stromelysins, matrilysin, membrane specific metalloproteinases, and other nonspecific metalloproteinases. These are protease family of enzymes, which cleaves extracellular matrix in general. In the brain, MMPs are important for the formation of neuronal remodeling, tissue matrix and blood-brain barrier activity and integrity. Inflammation, blood–brain barrier disruption, and extracellular remodeling have been suggested as the three major mechanisms for SVD; however, which one is the principal still remains controversial (Figure 4) [224,225]. Both inflammation and extracellular remodeling can modulate blood–brain function with a concurrent role of MMPs [20,225,226,227]. SVD was found to be associated with higher levels of the circulatory inhibitor of MMPs (TIMP-4) in the chronic phase whereas; in the acute phase, no such association was observed. The results indicate that endogenous TIMP-4 increased in response to the high level of MMPs activity and confirmed the role of MMPs in the progression of SVD [57].Single nucleotide polymorphism in MMP-2-1306 T/C is being considered a direct risk factor for the occurrence of isolated lacunar infarction, a form of SVD [228]. Out of six variant in MMP-9 gene, two loci interactions of rs3918242 and rs3787268 were associated with a higher risk for hemorrhagic transformation after ischemic stroke in Chinese population [229]. MMPs and their tissue inhibitors are inflammatory molecules that disrupt tight junctions and extracellular matrix, contributing to blood-brain barrier damage [230]. Normally, MMPs are present in the brain in latent forms, however, when they are induced and activated under conditions of hypoxia, they may disrupt the basal lamina and tight junctions of the cerebral blood vessels and degrade myelin basic protein [231,232,233]. MMPs induce the production of inflammatory cytokine N-acetyl proline-glycine-proline (ac-PGP) by the degradation of the extracellular matrix. ac-PGP is a neurotoxic mediator and causes sustain inflammation in the brain [234]. Gelatinase A (MMP-2) and gelatinase B (MMP-9) are reported to be active in hypoxic/ischemic injury. These MMPs induce infiltration of leukocytes and autoimmune inflammation in the brain [235]. It is evident that the deletion of MMP-9 can block the vascular pruning and hypoxia-induced angiogenesis. Vascular pruning requires post-hypoxia activation of MMP-9, which induces fragmentation of vascular laminin and claudin-5 (tight junction protein) [236]. The claudin-5 expression is regulated in glia by Sonic hedgehog signaling mediated transcription controlled byGli-1 [237]. Early expression of vascular endothelial growth factor may after ischemia, which then promotes further activation of MMPs [238] (Figure 4).

Micro bleeds and ischemia both are important characteristics of SVD at pathogenesis consequence levels. As discussed earlier, SVD has been found to be associated with WMA along with BBB dysfunction. In an MRI study, Egashira et al. have reported that MMP-9 is required for the white matter hyperintensity after sub-chronic hemorrhage [239]. SVD accounts for around one-fourth of all ischemic episodes and puts individuals at a two-time risk for these conditions with the concurrent role of MMPs [240]. Rempe et al. had demonstrated that glutamate an excitatory neurotransmitter could activate MMP-2 and MMP-9 in isolated brain capillaries [241]. A similar increase in glutamate surge occurs during reperfusion to the micro-ischemic area resulting in further enhancement of blood barrier leakages and causing microbleed. MMP-2/-9 mediated β-dystroglycan cleavage in ischemic conditions induces redistribution of aquaporin-4 in astrocytes and causes both cytotoxic and vasogenic edema [242,243,244]. These aquaporinsare sensitive to heavy metals such as Zn^2+^, Mn, Pb [245,246]. Manganese exerts neurotoxic effects through increased aquaporin-4 expression, which may cause astrocyte swelling [246]. Similarly, astrocytic aquaporin-4 is increased (40%) on lead exposure and decreases tight junction protein leading to the development of brain edema [247,248]. Contradictory to that, MMPs also promotes the migration of astrocytes and glial cells in the penumbra and decrease the infarct volume through the upregulation of extracellular-signal-regulated Kinase (ERK) [249]. Inhibition of MMP-12 has also been found to be useful in minimizing the reperfusion mediated BBB damage. The study also suggests that MMP-12 is involved in maintaining the blood–brain function. Further, MMP-12 has been demonstrated to have a downstream activation role to MMP-9 and other tissue-type plasminogen activator proteases [250]. Upstream to MMP-9, MMP-3 was also found to be involved in blood spinal cord barrier disruption [251].

The main target for MMPs is the BBB endothelium in the pathogenesis or progression of SVD. Initially, there is a release of proMT-MMP that is converted to MT-MPP in the presence of plasmin that further activates MMP-2, which is responsible for the reversible opening of BBB. This initiates the release of pro- and inflammatory cytokines like TNF-α, IL-β, NF-κB, etc., which further leads to activation of MMP-3 and MMP-9. All the consequences eventually lead to the delayed opening of BBB, which contributes to brain edema [252,253].

## 7. Role of Cellular Antioxidant Enzymes to Combat Metal-Induced CSVD

Cells have their own defense machinery to combat hostile oxidative environments such as Glutathione, superoxide dismutase, catalase, glutathione peroxidase, and additionally non-enzymatic ROS scavengers including vitamin C, vitamin E, β-carotene, and uric acid that has a specific mechanism to protect the cell from ROS attack during an oxidative event [254,255]. Commonly acknowledged hypothesis holds that as a result of an oxidative insult there may be an imbalance between the pro-oxidant and antioxidants. Significant data exist indicating that heavy metals exposure raises pro-oxidant markers (ROS, RNS, H_2_O_2_, MDA), conversely significantly diminishes the cellular antioxidant response [256]. In case of heavy metals poisoning, the superoxide level gets significantly elevated and to neutralize these highly reactive species, cell produces SOD enzyme that catalyzes ROS into hydrogen peroxide, which is also toxic to the cell. Further catalase breakdowns into water and oxygen. The excessive metal load decreases the antioxidant activity as well as expression in the cell, which may be due to the replacement of their functional cofactors [257,258]. In the early stages of vascular dysfunction, SOD and CAT activities get increased to protect and prevent lipid peroxidation; however, significant decline too has been observed in few cases amid the worsening of the disease [259]. Glutathione is highly expressed in every cell including endothelial to counter the toxic metabolites and also considered as a natural metal chelator thus preventing cell from the toxic effects of metal exposure [260]. Glutathione is a tri-peptide made from cysteine, glycine, and glutamic acid. The thiol group strongly attracts reactive species, and also forms a stable complex with the heavy metals thus reducing their toxic effects [261]. However, the higher exposure to heavy metals decreases glutathione level in the cell and leading to the deposition of these toxic metals inside the cell causing endothelium dysfunction and the impairment in BBB permeability [67,262]. Shimizu, et al. carried out a study on the cardiovascular patients and determined the total plasma glutathione concentrations between cases of cardiovascular disease. They reported that glutathione level was significantly lowered in the cerebral infarction, cerebral hemorrhage, case control, and also concluded reduced glutathione level a significant risk factor for CVD, especially for cerebral small vessel disease [263]. There is growing evidence that heavy metals exposure significantly decreases blood glutathione level [264,265]. Thus, it can be speculated that reduced glutathione could be the possible factor which contributes towards the etiology of heavy metal-induced small vessel diseases. There are reports that have highlighted the protective effect of GSH against metal-induced endothelial and BBB dysfunctions. Chang et al., reported that GSH treatment increased the biosynthesis of endogenous PGI_2,_ known to be a potent vasodilator and an inhibitor of platelet aggregation and thus protect from arsenic-induced endothelial cell death [266]. Song et al., too reported that glutathione treatment in rats reduces the cerebral infarct volume and cell death after ischemic injury and also explained that GSH administration improves the cell survival and preserves the disruption of BBB after ischemic injury [267].

## 8. Therapeutic Strategies

Chelation therapy could be a promising strategy to deal with the metal-induced physiological changes. It may decrease a heavy metal load from the body via fecal and urinary excretion thus reducing the responses of these toxic metals on human health [268]. Chelation therapy has shown promise in the diseases including AD, PD, autism, cancer, cardiovascular disease, etc. [269,270]. Chelating agents not only reduces raised metal concentration but some thiol based chelators, such as meso 2,3-dimercaptosuccinic acid (DMSA), 2,3-dimercaptopropane 1-sulfonate (DMPS), D-pencillamine (DPA), and Monoisoamyldimercaptosuccinic acid (MiADMSA) (Table 6) also have ROS neutralizing ability that can additionally diminish the augmented oxidative stress and further preventing cellular damage from oxidative insult [271]. Available clinical evidences show the protective effect of EDTA against metal-induced endothelial dysfunctions in cardiovascular disease [272,273,274]. It has been reported that atherosclerotic plague retains the toxic metal and further promotes vascular dysfunction. To counter such situation we suggest use of an adjuvant during chelation therapy, which could be a promising approach [275,276]. Sompamit et al., reported the protective effect of meso-2,3-dimercaptosuccinic acid against cadmium-induced vascular dysfunction in mice and suggested that DMSA treatment reduced systemic metal load and also restored the augmented oxidative stress thus ameliorating the vascular dysfunction [277]. Evidences also suggest that chelation therapy could be an effective tool to reduce cerebral amyloid angiopathy by reducing copper load from the brain. [103]. In vivo study demonstrated that the protective effect of tetrathiomolybdate (TTM), a copper chelator, in Tg2576 mice. The study reported that TTM therapy significantly reduces fibrillar amyloid in both the CAA vessels and parenchymal plaques. Further studies are required to broaden the therapeutic applicability of chelation therapy in amyloid angiopathy [96]. Apart from the chelation therapy, antioxidant may also be effective and must be tried in metal-induced cerebral vascular diseases. Antioxidantsare the molecules that may inhibit or delay the oxidation of the substrate at a lower dose. An antioxidant molecule can elicit responses through number of mechanism including ROS scavenging, by increasing the level of cellular antioxidant enzymes, reducing lipid peroxidation, and by maintaining the redox process by reducing the augmented redox metal level (Cu and Fe). There are antioxidants, which provide additional benefit like the ability to chelate heavy metals (Pb, As, Hg, etc.). They include antioxidant like lipoic acid, resveratrol, curcumin, which have shown promising results in number of in-vitro and in-vivo studies. However, the use of antioxidant therapy in cerebrovascular disease still needs validation. Indeed, several promising approaches failed in clinical trials [11].

## 9. Possible Measures to Avoid Heavy Metals Exposure

Humans have been utilizing heavy metals since antiquity; therefore, complete avoidance of the use of heavy metals sounds paradoxical. However, the limited use of heavy metals and alternatives might be a good approach to limit their exposure to humans, such as:

- Controls of the heavy metal level in the water and food [278];
- Alternatives to dental amalgam;
- Alternative use of heavy metal-based agrochemicals such as copper oxychloride;
- Alternative use of copper plumbing;
- Stop smoking because cigarette smoke contains cadmium that can be absorbed through the lungs [279];
- Pay attention to local fish advisories regarding mercury levels and also try to limit your consumption of larger fish because they live long and absorb more mercury from the sea [280];
- Wear masks and protective clothing to avoid occupational exposure [281].

## 10. Conclusions and Future Direction

Cerebral Small Vessel Disease is a major health threat; several risk factors, including genetic, co-morbid complications, and environmental factors contribute to the pathogenesis or exacerbate the complications. From the evidence outlined in this review, it is apparent that exposure to heavy metals may lead to the abnormalities in small vessels and endothelium dysfunction associated with CSVD. Vascular endothelial cells are highly vulnerable to heavy metals as they are directly exposed to heavy metals present in the circularity bloodstream. Heavy metals are known to produce ROS that may create the imbalance between pro-oxidant and antioxidants, besides, generation of superoxide reduce the NO bioavailability in the vascular endothelial cells. ROS triggers inflammatory response through the NF-κB mediated pathway. Copper also induces the amyloid precipitation in the brain, which is the leading cause of vascular angiopathy and dementia. More detailed investigations are required to understand the roles of heavy metals exposure and small vessel diseases. It will be imperative to elucidate how heavy metals trigger the up- and downstream molecular pathways, particularly oxidative instigated. Although treatment with chelating agents and antioxidants have shown some promise against heavy metal-induced vascular and neurological impairments, they have some shortcomings and side effects, particularly their binding to other essential metals within the system, which significantly reduce their efficacy. In terms of novel therapeutic options for patients with CSVD and associated disorders, a combination therapy using an adjuvant and a chelating agent, which may be useful in controlling toxic metal load and oxidative injury (associated with the whole pathogenesis) may represent an exclusive strategy to tackling metal-induced cerebral small vessels disease. Further studies are also required in this area to describe the toxic limits of heavy metal, precise molecular mechanism, and therapeutic strategies.

## Figures and Tables

**Table 1:** Experimental evidences showing the role of Pb in microvascular endothelial and blood–brain barrier (BBB) dysfunctions.

System	Concentration	Exposure Duration	Toxicity	Ref.
In-vitro (hCMEC/D3 Cell line	25–200 µM Pb	48 h	Abnormalities in hCMEC/D3 cells monolayer via an oxidative stress-mediated pathway. Oxidative stress key contributing mechanism for BBB dysfunction.	[67]
In-vivo male Wistar rats	200 mg/L lead acetate Drinking water	3 Months	Blood – brain dysfunction. Endothelial cells exhibit higher pinocytotic activity and also opening of inter-endothelial tight junctions.	[75]
In-vitro RBE4 cell line	10^−5^ M and 10^−6^ M lead acetate at	2 h, 4 h, 8 h, 16 h, and 24 h	Reduces occludin, claudin 5, ZO1, and ZO2 protein expression. Increases the permeability of the neurotoxicant from BBB. Decreases the protein expressions that maintain the tight junction.	[76]
In-vitro epithelial Z310 cells	5 and 10 μM Pb	Pre and post exposure	Decreased occludin protein leading to BBB dysfunction.	[77]
In-vivo Sprague-Dawley dams	4% lead carbonate via feed	5, 10, 15 days	Vasogenic edema formation through the Flk1–dependent pathway. Microvascular hemorrhage development and BBB permeability to albumin. Encephalopathy by VEGF-independent pathway.	[78]
In-vivo Male SD rats	100, 200, 300 PPM/mL Drinking water	eight weeks	Disruption of BBB due to lower expression of occludin protein that might be caused by GRP78 expressions leading to Src activation and altering occluding expression.	[79]
In-vivo Male SD rats	50 mg/kg Pb acetate i.p., injection	24 h	Increased Aβ levels in the BCP due to abnormal Aβ homeostasis at the CP mediated by LRP1 via PKC-δ pathway.	[80]
Primary culture brain microvessels isolated from 6-day-old rat pups	1 μM lead	0–60 min	Microvessel toxicity by affecting protein kinase C activity that possible underlying mechanism Pb induced immature brain microvessels.	[81]
In vitro C6 glia cells and ECV304	2.5, 5, 10 μM Pb	6, 12, 24, 48 h	Increased MMP-2 and MMP-9 suggesting BBB dysfunction. Reduced expressions of ZO-1 and occludin.	[82]
In- vivo Male Sprague–Dawley rats	342 μg Pb/mL as Pb acetate	Once every other day 6 weeks	Pb accumulation causes BBB dysfunction. Decreased expression of occluding.	[83]

Abbreviations: hCMEC/D3: Human Cerebral Microvascular Endothelial Cell Line, BBB: Blood brain barrier, Pb: Lead, RBE4: Rat Brain Endothelial 4, Flk1: Fetal Liver Kinase 1, VEGF: Vascular Endothelial Growth Factor, GRP78: Glucose Regulated Protein 78, LRP1: Low-density lipoprotein receptor-related protein-1, PKC-δ: Protein kinase C delta, MMP: Matrix metallopeptidase, ZO: Zona Occludens, BCP: Bilateral Cerebral Plexus, CP: Choroid Plexus.

**Table 2:** Experimental evidences showing the Cu role in microvascular endothelial and BBB dysfunctions.

System	Concentration	Exposure Duration	Toxicity	Ref.
In-vitro rBMECs cells	1.5–50 μg/mL (Cu nanoparticles)	0–8 h	Cytotoxic at the higher concentration. Increase TNF-a, IL-1b and IL-2 time-dependently.	[94]
Primary culture (SPF Wistar neonate rats) Brain microvascular endothelial cells (BMECS)	30–300 μM (Cucl_2_)	12 h	Cu at 30–120 μM increased cell viability due to increased antioxidant activity in the cell such as CAT and SOD. Cu at 180–300 μM showed a cytotoxic effect and oxidative stress. Alter the claudin protein expression.	[100]
In-vitro HUVEC, HMEC-L, and HIAEC cells	10 to 50 μM (Cucl_2_)	12 h	Cu exposure increases inflammatory responses driven by the NF-κB a PI3-kinase/Akt pathway. Higher IL-8 expression.	[101]
In-vivo Sprague Dawley rats	IP 50 mg/kg IV 30 mg/kg Cortical superfusion (20 µg/10 µL) (Cu nanoparticles)	24 h	Cu nanoparticles-induced BBB disruption. Brain edema formation.	[102]
AD patients	-	-	Cu was found to be deposited in the arteriolar tree in CAA. Vascular fragility observed.	[103]
In-vivo C57BL6 mice	1 mg/L (Cucl_2_ + cholesterol As a risk factor) Drinking water	4 weeks	BBB disruption and, cerebral bleedings. Cognitive dysfunctions and anxiety observed in mice.	[104]
In-vivo 3xTg-AD	250 ppm Cu sulfate (CuSO_4_) Drinking water	3 or 9 months	Cu exposure displayed AD pathology through amyloid and tau mediated pathway.	[105]
In-vivo Male Wistar rats	10 µg/mL (Cucl_2_)	1 h	Acute Cu exposure decrease the vascular functions. Oxidative stress generation through the iNOS pathway.	[106]
In- vitro Bovine aortic endothelial cells (BAECs)	0–500 μg/mL Cu_2_O	12 h	Cu increases ROS generation and autophagy by an AMPK pathway. Cu_2_O crystals elicit endothelial cell death through autophagy.	[107]
In- vitro human aortic endothelial cells (HAECs)	100 μM Cupric sulfate	0–16 h	Cu increased VCAM-1, ICAM-1, and MCP-1 expression. Activate NF-κB and AP-1 signaling.	[108]

Abbreviations: BMECs: Brain microvascular endothelial cells, Cu: Copper, TNF: Tumor necrosis factor, IL: Interleukin, SPF: Specific-pathogen-free, Cucl_2_: Copper(II) chloride, CAT: Catalase, SOD: Super oxide dismutase, HUVEC: Human umbilical vein endothelial cell, HMEC: Human man mammary epithelial cells, HIAEC: Human lilac endothelial cells, NF-κB: Nuclear factor kappa B, PI3:Phosphoinositide 3-kinase, Akt: Protein kinase B, AD: Alzheimer’s disease, iNOS: Inducible nitric oxide synthase, CAA: Cerebral amyloid angiopathy, VCAM: Vascular cell adhesion protein, ICAM: Intercellular Adhesion Molecule, MCP: Monocyte chemo attractant protein, AP: Activator protein, ROS: Reactive Oxygen Species, AMPK: 5’ Adenosine monophosphate-activated protein kinase.

**Table 3:** Experimental evidences showing the role of Hg in the microvascular endothelial and BBB dysfunctions.

System	Concentration	Exposure Duration	Toxicity	Ref.
In-vivo normotensive Wistar rats	HgCl_2_ (first dose 4.6 μg/kg, subsequent dose 0.07 μg/kg/day, im to cover daily loss)	30 days	Elevates ROS generation that causes vascular reactivity and decreases the NO bioavailability.	[115]
In-vitro HUVECs	(1.0–5.0 microM) MeHg	24 h	Dose-dependent cytotoxic effect on the HUVECs cell. Disruption of endothelial functions.	[125]
In-vitro Human brain micro-vascular cells	(1, 2, 3 µM MeHg) (2 µM HgCl_2_)	24 h	Increased LDH leakage from the cells. Reduced proliferation of endothelial cells.	[117]
In-vivo male Wistar rats	20-ppm MeHg Drinking water	4 weeks	BBB damage through the upregulation of VEGF expression.	[126]
In-vivo Sprague-Dawley rats	1.0 mg/kg Mercuric bichloride Subcutaneous	30 min, 1 h, 6 h, 12 h, 24 h, and 1 week after the mercury administration	Endothelial and glial impairment. BBB dysfunction.	[127]
In-vitro Human brain microvascular endothelial cells	(1, 2, 3, and 5 µM) MeHg	24 h	Increased release of PGI2 and Cox-2 from the endothelial cells and pericytes. Modulate the p38 and MAPK pathways.	[128]
In- vitro Bovine aortic endothelial cells (BAECs)	1 μM MeHg	1, 3 or 6 h, 24 h	Disrupt mitochondrial membrane potential. Activated NADPH-oxidase pathway and caused endothelial dysfunction.	[129]
In-vitro Human brain microvascular endothelial cells	1, 2, 3 µM MeHg	24 h	Decreased expression of FGF-2 protein.	[119]
In-vitro PC12 cells	0, 10, 100, 1000 nM HgCl_2_	48 h	Increased APP synthesis and amyloid beta accumulation.	[130]

Abbreviations: HgCl_2_- Mercury (II) chloride, ROS: Reactive oxygen species, NO: Nitric oxide, HUVECs: Human umbilical vein endothelial cells, MeHg: methylmercury, LDH: Lactate dehydrogenase, BBB: Blood–brain barrier, VEGF: Vascular endothelial growth factor, PGI2: Prostacyclin or prostaglandin I2, Cox-2: Cyclooxygenase-2, MAPK: Mitogen-activated protein kinase, BAECs: Bovine aortic endothelial cells, NADPH: Nicotinamide adenine dinucleotide phosphate hydrogen, FGF-2: Fibroblast growth factor, APP: Amyloid precursor protein.

**Table 4:** Experimental evidences showing the As role in microvascular endothelial and BBB dysfunctions.

System	Concentration	Exposure Duration	Toxicity	Ref.
Invitro HUVECs	5 µM arsenic trioxide	24 h	Impaired NO production. Endothelial Activation and Apoptosis, Oxidative stress.	[164]
In vitro SVEC4-10	7.5 µM arsenic trioxide	4–6 h	Cytotoxicity and oxidative stress. Increase NF-κB, and decreased HO-1 and VEGF expression.	[165]
In vitro SVEC4-10	5 and 7.5 µM arsenic trioxide	4–6 h	ER pathway mediated endothelial cytotoxicity. Upregulation of Apoptosis cascade.	[166]
In vitro HAEC	1, 10, 100, and 1000 ng/mL Arsenic trioxide	5–72 h	Endothelial gap junctions are downregulated. Decreases eNOS protein and NO availability.	[167]
In vitro HUVECs	1–5 µM Sodium arsenite	24 h	Endothelial activation. Increases VEGF expression.	[168]
Invivo Kunming mice	0.15 mg 1.5 mg 15 mg arsenic trioxide/L Drinking water	whole lactation period (postnatal day 42)	The decrease in the mRNA expression levels of TJ proteins (Occludin, Claudin, ZO-1 and ZO-2) and Occludin protein. Induces autophagy by inhibiting PI3K/Akt/mTOR signaling pathway.	[169]
Invivo Wistar rats	100 ppm Sodiumarsenite Drinking water	60 days	Induces Oxidative stress, and memory impairment. Induces endothelial dysfunction and dementia.	[170]
Invivo Wistar rats	4–5 mg/kg/ day arsenite Drinking water	Gestation, lactation and until 4 months of age	Lower response to NMDA receptor stimulation. Reduction of NOS activity and decreased levels of nitrites.	[154]
54 arsenicosis patients	-	-	Impairment of the NO/cGMP pathway in both males and females.	[171]

Abbreviations: HUVECs: Human umbilical vein endothelial cells, NO: Nitric oxide, NF-κB: Nuclear factor kappa- B, HO-1: Heme oxygenase-1, VEGF: Vascular endothelial growth factor, ER: Endoplasmic reticulum, HAEC: Human aortic endothelial cells, eNOS: Endothelial NOS, mRNA: Messenger ribonucleic acid, TJ proteins: Tight junction proteins, ZO-1: Zonula occludens-1, ZO-2: Zonula occludens-2, PI-3K: Phosphatidylinositol 3-kinase, Akt: protein kinase B, mTOR: Mammalian target of rapamycin, NMDA: N-methyl-D-aspartate receptor, NOS: Nitric-oxide Synthase, cGMP: Cyclic guanosine monophosphate.

**Table 5:** Experimental evidences showing role of Cd role on brain oxidative stress, apoptosis and endothelial dysfunctions.

System	Concentration	ExposureDuration	Toxicity	Ref.
Zebrafish	1 mg/L	24 h and 96 h	Altered redox balance in the brain at both gene and protein levels. Increased inflammatory cytokines.	[175]
Zebrafish embryos	9 µM 1.0 mg/L	24 h 7 days	Change in the morphology of glial and neuronal cells. Change in the brain mitochondrial morphology.	[176] [177]
Mice	3 mg/L Drinking water	20 weeks	Brain function impairment. Olfactory function and memory impairments.	[192]
Rats	3 mg/kg Orally	28 days	Oxidative imbalance and DNA damage in the brain. Upregulate dShh signaling pathway. Loss of cerebellum structural integrity and motor function.	[193]
Rats	5 mg/kg bodyweight Orally	21 days	Increased brain cholinesterase, MAO, Na(+)/K(+)-ATPase activities.	[179]
Rabbits	CdCl_2_ 3 mg/kg × bw Orally	30 days	Downregulation of Metallothionein along with antioxidant genes.	[183]
Rats	32.5 ppm Drinking water	2, 3- and 4-month	Decreased density of dendritic spines. Increased caspase-3 and 9 immunoreactivity.	[184]
Juvenile mice	3.74 mg/kg Orally	10 days	Hypotrophy and alteration in microstructure and ultrastructure alterations.	[185]
Rats	4 mg/kg bw i.p. Route	Single-dose	A decrease in endothelial barrier antigen expression. Astrocytic deformation. Blurred white and gray matter cytoplasm.	[189]

Abbreviations: NO: Nitric oxide, DNA: Deoxyribonucleic acid, MAO: Monoamine oxidase, Na⁺/K⁺-ATPase: Sodium–potassium adenosine triphosphatase, CdCl2: Cadmium chloride, ppm: parts per million, shh: sonic hedgehog.

**Table 6:** Heavy Metal Chelators.

Compound Name	Abbreviation	Molecular Formula	Structure
Calcium Disodium Ethylenediamine Tetra acetic Acid	CaNa_2_EDTA	C_10_H_12_CaN_2_Na_2_O_8_	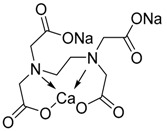
British Anti-Lewisite or 2,3- Dimercaprol	BAL	C_3_H_8_OS_2_	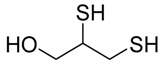
Tetrathiomolybdate	TM	MoS_4_^2-^	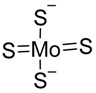
D-Pencillamine	DPA	C_5_H_11_NO_2_S	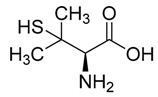
meso-2,3-dimercaptosuccinic acid	DMSA	C_4_H_6_O_4_S_2_	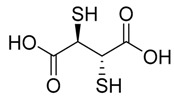
Sodium 2,3 Dimercaptopropane-l-Sulphonate	DMPS	C_3_H_7_NaO_3_S_3_	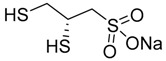
Monoisoamyldimercaptosuccinic acid	MiADMSA	C_9_H_16_O_4_S_2_	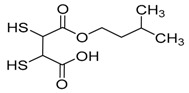

## Abbreviations

AD	Alzheimer’s disease
As	Arsenic
ATP	Adenosine triphosphate
CAA	Cerebral amyloid angiopathy
CAT	Catalase
Cd	Cadmium
CNS	Central Nervous System
CSVD	Cerebral Small Vessel Disease
CTR1	High-affinity copper uptake protein 1
CVD	Cardiovascular disease
Cu	Copper
DMSA	2,3-dimercaptosuccinic acid
DNA	Deoxyribonucleic acid
EC	Endothelial cells
EDTA	Ethylenediaminetetraacetic acid,
GPx	Glutathione Peroxidase
GSH	Glutathione
Hg	Mercury
H_2_O_2_	Hydrogen Peroxide
IL	Interleukin
IkK	IκB kinase
MDA	Malondialdehyde
MeHg	Methyl mercury
MMPs	Matrix metalloproteinase
NADPH	Nicotinamide adenine dinucleotide phosphate
NF-κB	Nuclear factor-kappa B3
NO	Nitric oxide
Pb	Lead
ROS	Reactive oxygen species
SD rat	Sprague Dawley
SOD	Super Oxide Dismutase
TNF-α	Tumor necrosis factor-α
TTM	Tetrathiomolybdate
VEGF	Vascular endothelial growth factor
WD	Wilsons Disease
eNOS	Endothelial nitric oxide synthase
hCMEC/D3	Human brain microvascular endothelial cells

## References

[1] Joutel A., Faraci F.M. (2014). Cerebral small vessel disease: Insights and opportunities from mouse models of collagen IV-related small vessel disease and cerebral autosomal dominant arteriopathy with subcortical infarcts and leukoencephalopathy. Stroke.

[2] Pantoni L. (2010). Cerebral small vessel disease: From pathogenesis and clinical characteristics to therapeutic challenges. Lancet Neurol..

[3] Das A.S., Regenhardt R.W., Vernooij M.W., Blacker D., Charidimou A., Viswanathan A. (2019). Asymptomatic Cerebral Small Vessel Disease: Insights from Population-Based Studies. J. Stroke.

[4] Cuadrado-Godia E., Dwivedi P., Sharma S., Ois Santiago A., Roquer Gonzalez J., Balcells M., Laird J., Turk M., Suri H.S., Nicolaides A. (2018). Cerebral Small Vessel Disease: A Review Focusing on Pathophysiology, Biomarkers, and Machine Learning Strategies. J. Stroke.

[5] Giau V.V., Bagyinszky E., Youn Y.C., An S.S.A., Kim S.Y. (2019). Genetic Factors of Cerebral Small Vessel Disease and Their Potential Clinical Outcome. Int. J. Mol. Sci..

[6] Khan U., Porteous L., Hassan A., Markus H.S. (2007). Risk factor profile of cerebral small vessel disease and its subtypes. J. Neurol. Neurosurg. Psychiatry.

[7] Prozialeck W.C., Edwards J.R., Nebert D.W., Woods J.M., Barchowsky A., Atchison W.D. (2008). The vascular system as a target of metal toxicity. Toxicol. Sci..

[8] Chowdhury R., Ramond A., O’Keeffe L.M., Shahzad S., Kunutsor S.K., Muka T., Gregson J., Willeit P., Warnakula S., Khan H. (2018). Environmental toxic metal contaminants and risk of cardiovascular disease: Systematic review and meta-analysis. BMJ.

[9] Jan A.T., Azam M., Siddiqui K., Ali A., Choi I., Haq Q.M.R. (2015). Heavy Metals and Human Health: Mechanistic Insight into Toxicity and Counter Defense System of Antioxidants. Int. J. Mol. Sci..

[10] Tchounwou P.B., Yedjou C.G., Patlolla A.K., Sutton D.J. (2012). Heavy metal toxicity and the environment. EXS.

[11] Carvalho C., Moreira P.I. (2018). Oxidative Stress: A Major Player in Cerebrovascular Alterations Associated to Neurodegenerative Events. Front. Physiol..

[12] Flora S.J.S., Mittal M., Mehta A. (2008). Heavy metal induced oxidative stress & its possible reversal by chelation therapy. Indian J. Med. Res..

[13] Nita M., Grzybowski A. (2016). The role of the reactive oxygen species and oxidative stress in the pathomechanism of the age-related ocular diseases and other pathologies of the anterior and posterior eye segments in adults. Oxid. Med. Cell Longev..

[14] Mlynek V., Skoczynska A. (2005). The proinflammatory activity of cadmium. Postepy. Hig. Med. Dosw.

[15] Li Q., Yang Y., Reis C., Tao T., Li W., Li X., Zhang J.H. (2018). Cerebral Small Vessel Disease. Cell Transplant..

[16] Wardlaw J.M., Smith E.E., Biessels G.J., Cordonnier C., Fazekas F., Frayne R., Lindley R.I., O’Brien J.T., Barkhof F., Benavente O.R. (2013). Neuroimaging standards for research into small vessel disease and its contribution to ageing and neurodegeneration. Lancet Neurol..

[17] Poggesi A., Pasi M., Pescini F., Pantoni L., Inzitari D. (2016). Circulating biologic markers of endothelial dysfunction in cerebral small vessel disease: A review. J. Cereb. Blood Flow Metab..

[18] Rouhl R.P., van Oostenbrugge R.J., Damoiseaux J.G., Debrus-Palmans L.L., Theunissen R.O., Knottnerus I.L., Staals J.E., Delanghe J.R., Tervaert J.W., Lodder J. (2009). Haptoglobin phenotype may alter endothelial progenitor cell cluster formation in cerebral small vessel disease. Curr. Neurovasc. Res..

[19] Vernooij M.W., van der Lugt A., Ikram M.A., Wielopolski P.A., Niessen W.J., Hofman A., Krestin G.P., Breteler M.M. (2008). Prevalence and risk factors of cerebral microbleeds: The Rotterdam Scan Study. Neurology.

[20] Wardlaw J.M., Sandercock P.A., Dennis M.S., Starr J. (2003). Is breakdown of the blood-brain barrier responsible for lacunar stroke, leukoaraiosis, and dementia?. Stroke.

[21] Fassbender K., Bertsch T., Mielke O., Mühlhauser F., Hennerici M. (1999). Adhesion molecules in cerebrovascular diseases: Evidence for an inflammatory endothelial activation in cerebral large-and small-vessel disease. Stroke.

[22] Lum H., Roebuck K.A. (2001). Oxidant stress and endothelial cell dysfunction. Am. J. Physiol. Cell Physiol..

[23] Sena C.M., Louro T., Matafome P., Nunes E., Monteiro P., Seica R. (2009). Antioxidant and vascular effects of gliclazide in type 2 diabetic rats fed high-fat diet. Physiol. Res..

[24] Sena C.M., Matafome P., Louro T., Nunes E., Fernandes R., Seiça R.M. (2011). Metformin restores endothelial function in aorta of diabetic rats. Br. J. Pharmacol..

[25] Norlander A.E., Madhur M.S., Harrison D.G. (2018). The immunology of hypertension. J. Exp. Med..

[26] Zhou N., Lee J.J., Stoll S., Ma B., Costa K.D., Qiu H. (2017). Rho Kinase Regulates Aortic Vascular Smooth Muscle Cell Stiffness Via Actin/SRF/Myocardin in Hypertension. Cell Physiol. Biochem..

[27] Rempe R.G., Hartz A.M.S., Bauer B. (2016). Matrix metalloproteinases in the brain and blood-brain barrier: Versatile breakers and makers. J. Cereb. Blood Flow Metab..

[28] Deng J., Zhang J., Feng C., Xiong L., Zuo Z. (2014). Critical role of matrix metalloprotease-9 in chronic high fat diet-induced cerebral vascular remodelling and increase of ischaemic brain injury in mice. Cardiovasc. Res..

[29] Kelly P.J., Morrow J.D., Ning M., Koroshetz W., Lo E.H., Terry E., Milne G.L., Hubbard J., Lee H., Stevenson E. (2008). Oxidative stress and matrix metalloproteinase-9 in acute ischemic stroke: The Biomarker Evaluation for Antioxidant Therapies in Stroke (BEAT-Stroke) study. Stroke.

[30] Dong X., Song Y.-N., Liu W.-G., Guo X.-L. (2009). Mmp-9, a potential target for cerebral ischemic treatment. Curr. Neuropharmacol..

[31] Haffner C., Malik R., Dichgans M. (2016). Genetic factors in cerebral small vessel disease and their impact on stroke and dementia. J. Cereb. Blood Flow Metab..

[32] Marini S., Anderson C.D., Rosand J. (2020). Genetics of cerebral small vessel disease. Stroke.

[33] Staals J., Makin S.D.J., Doubal F.N., Dennis M.S., Wardlaw J.M. (2014). Stroke subtype, vascular risk factors, and total MRI brain small-vessel disease burden. Neurology.

[34] Bushara S.O., Noor S.K., Ibraheem A.A.H., Elmadhoun W.M., Ahmed M.H. (2016). Prevalence of and risk factors for hypertension among urban communities of North Sudan: Detecting a silent killer. J. Fam. Med. Prim. Care..

[35] Vijayan M., Reddy P.H. (2016). Stroke, Vascular Dementia, and Alzheimer’s Disease: Molecular Links. J. Alzheimers Dis..

[36] Martini S.R., Williams S.R., Moretti P., Woo D., Worrall B.B. (2015). A molecular/genetic approach to cerebral small-vessel disease: Beyond aging and hypertension. Brain Circ..

[37] Namba T., Nolte C.T., Jackrel J., Grob D. (1971). Poisoning due to organophosphate insecticides: Acute and chronic manifestations. Am. J. Med..

[38] Lin C.-H., Hsu Y.-T., Yen C.-C., Chen H.-H., Tseng C.-J., Lo Y.-K., Chan J.Y.H. (2018). Association between heavy metal levels and acute ischemic stroke. J. Biomed. Sci..

[39] Shinkai Y., Kaji T. (2012). Cellular defense mechanisms against lead toxicity in the vascular system. Biol. Pharm. Bull..

[40] Kim J.-H., Byun H.-M., Chung E.-C., Chung H.-Y., Bae O.-N. (2013). Loss of Integrity: Impairment of the Blood-brain Barrier in Heavy Metal-associated Ischemic Stroke. Toxicol. Res..

[41] Kumar J., Sathua K.B., Flora S.J.S. (2019). Chronic copper exposure elicits neurotoxic responses in rat brain: Assessment of 8-hydroxy-2-deoxyguanosine activity, oxidative stress and neurobehavioral parameters. Cell. Mol. Biol..

[42] Gattringer T., Eppinger S., Pinter D., Pirpamer L., Berghold A., Wunsch G., Ropele S., Wardlaw J.M., Enzinger C., Fazekas F. (2015). Morphological MRI characteristics of recent small subcortical infarcts. Int. J. Stroke.

[43] Malayeri A.A., El Khouli R.H., Zaheer A., Jacobs M.A., Corona-Villalobos C.P., Kamel I.R., Macura K.J. (2011). Principles and applications of diffusion-weighted imaging in cancer detection, staging, and treatment follow-up. Radiographics.

[44] Huisman T.A. (2003). Diffusion-weighted imaging: Basic concepts and application in cerebral stroke and head trauma. Eur. J. Radiol..

[45] Baliyan V., Das C.J., Sharma R., Gupta A.K. (2016). Diffusion weighted imaging: Technique and applications. World J. Radiol..

[46] Okazaki S., Hornberger E., Griebe M., Gass A., Hennerici M.G., Szabo K. (2015). MRI Characteristics of the Evolution of Supratentorial Recent Small Subcortical Infarcts. Front. Neurol..

[47] Aribisala B.S., Valdes Hernandez M.C., Royle N.A., Morris Z., Munoz Maniega S., Bastin M.E., Deary I.J., Wardlaw J.M., Bastin M.E., Deary I.J. (2013). Brain atrophy associations with white matter lesions in the ageing brain: The Lothian Birth Cohort 1936. Eur. J. Radiol..

[48] Wardlaw J.M., Valdes Hernandez M.C., Munoz-Maniega S. (2015). What are white matter hyperintensities made of? Relevance to vascular cognitive impairment. J. Am. Heart Assoc..

[49] De Silva T.M., Miller A.A. (2016). Cerebral Small Vessel Disease: Targeting Oxidative Stress as a Novel Therapeutic Strategy?. Front. Pharmacol..

[50] Schmidt R., Grazer A., Enzinger C., Ropele S., Homayoon N., Pluta-Fuerst A., Schwingenschuh P., Katschnig P., Cavalieri M., Schmidt H. (2011). MRI-detected white matter lesions: Do they really matter?. J. Neural Transm..

[51] Kim H.J., Im K., Kwon H., Lee J.M., Ye B.S., Kim Y.J., Cho H., Choe Y.S., Lee K.H., Kim S.T. (2015). Effects of amyloid and small vessel disease on white matter network disruption. J. Alzheimers Dis..

[52] Fazekas F., Kleinert R., Roob G., Kleinert G., Kapeller P., Schmidt R., Hartung H.P. (1999). Histopathologic analysis of foci of signal loss on gradient-echo T2*-weighted MR images in patients with spontaneous intracerebral hemorrhage: Evidence of microangiopathy-related microbleeds. Am. J. Neuroradiol..

[53] Patel B., Lawrence A.J., Chung A.W., Rich P., Mackinnon A.D., Morris R.G., Barrick T.R., Markus H.S. (2013). Cerebral microbleeds and cognition in patients with symptomatic small vessel disease. Stroke.

[54] Shi Y., Wardlaw J.M. (2016). Update on cerebral small vessel disease: A dynamic whole-brain disease. Stroke Vasc. Neurol..

[55] Cordonnier C., Potter G.M., Jackson C.A., Doubal F., Keir S., Sudlow C.L., Wardlaw J.M., Al-Shahi Salman R. (2009). improving interrater agreement about brain microbleeds: Development of the Brain Observer MicroBleed Scale (BOMBS). Stroke.

[56] Zhang Y., Dong H., Seeburg D.P., Wojtkiewicz G.R., Waterman P., Pulli B., Forghani R., Ali M., Iwamoto Y., Swirski F.K. (2019). Multimodal Molecular Imaging Demonstrates Myeloperoxidase Regulation of Matrix Metalloproteinase Activity in Neuroinflammation. Cell. Mol. Neurobiol..

[57] Arba F., Piccardi B., Palumbo V., Giusti B., Nencini P., Gori A.M., Sereni A., Nesi M., Pracucci G., Bono G. (2019). Small Vessel Disease Is Associated with Tissue Inhibitor of Matrix Metalloproteinase-4 After Ischaemic Stroke. Transl. Stroke Res..

[58] Muller M., Appelman A.P., van der Graaf Y., Vincken K.L., Mali W.P., Geerlings M.I. (2011). Brain atrophy and cognition: Interaction with cerebrovascular pathology?. Neurobiol. Aging.

[59] Berlow Y.A., Wells W.M., Ellison J.M., Sung Y.H., Renshaw P.F., Harper D.G. (2010). Neuropsychiatric correlates of white matter hyperintensities in Alzheimer’s disease. Int. J. Geriatr. Psychiatry.

[60] Pande M., Flora S.J.S. (2002). Lead induced oxidative damage and its response to combined administration of α-lipoic acid and succimers in rats. Toxicology.

[61] Kalia K., Flora S.J.S. (2005). Strategies for safe and effective therapeutic measures for chronic arsenic and lead poisoning. J. Occup. Health.

[62] Flora S.J.S., Flora G., Saxena G. (2006). Environmental occurrence, health effects and management of lead poisoning. Lead.

[63] Flora S.J.S., Saxena G., Mehta A. (2007). Reversal of lead-induced neuronal apoptosis by chelation treatment in rats: Role of reactive oxygen species and intracellular Ca2+. J. Pharmacol. Exp. Ther..

[64] Rabinowitz M.B., Wetherill G.W., Kopple J.D. (1976). Kinetic analysis of lead metabolism in healthy humans. J. Clin. Invest..

[65] Mason L.H., Harp J.P., Han D.Y. (2014). Pb neurotoxicity: Neuropsychological effects of lead toxicity. Biomed. Res. Int..

[66] Sanders T., Liu Y., Buchner V., Tchounwou P. (2009). B. Neurotoxic effects and biomarkers of lead exposure: A review. Rev. Environ. Health.

[67] .Tobwala S., Wang H.-J., Carey J.W., Banks W.A., Ercal N. (2014). Effects of lead and cadmium on brain endothelial cell survival, monolayer permeability, and crucial oxidative stress markers in an in vitro model of the blood-brain barrier. Toxics.

[68] Pachauri V., Saxena G., Mehta A., Mishra D., Flora S.J.S. (2009). Combinational chelation therapy abrogates lead-induced neurodegeneration in rats. Toxicol. Appl. Pharmacol..

[69] Pachauri V., Dubey M., Yadav A., Kushwaha P., Flora S. (2012). Monensin potentiates lead chelation efficacy of MiADMSA in rat brain post chronic lead exposure. Food Chem. Toxicol..

[70] Kevil C.G., Okayama N., Trocha S.D., Kalogeris T.J., Coe L.L., Specian R.D., Davis C.P., Alexander J.S. (1998). Expression of zonula occludens and adherens junctional proteins in human venous and arterial endothelial cells: Role of occludin in endothelial solute barriers. Microcirculation.

[71] Trocha S.D., Kevil C.G., Mancini M.C., Alexander J.S. (1999). Organ preservation solutions increase endothelial permeability and promote loss of junctional proteins. Ann. Surg..

[72] Liu X., Zheng G., Wu Y. (2013). Lead exposure results in hearing loss and disruption of the cochlear blood-labyrinth barrier and the protective role of iron supplement. Neurotoxicology.

[73] Cecil K.M., Brubaker C.J., Adler C.M., Dietrich K.N., Altaye M., Egelhoff J.C., Wessel S., Elangovan I., Hornung R., Jarvis K. (2008). Decreased brain volume in adults with childhood lead exposure. PLoS Med..

[74] Kim Y.J., Kwon H.K., Lee J.M., Cho H., Kim H.J., Park H.K., Jung N.-Y., San Lee J., Lee J., Jang Y.K. (2016). Gray and white matter changes linking cerebral small vessel disease to gait disturbances. J. Neurol..

[75] Strużyńska L., Walski M., Gadamski R., Dabrowska-Bouta B., Rafałowska U. (1997). Lead-induced abnormalities in blood-brain barrier permeability in experimental chronic toxicity. Mol. Chem. Neuropathol..

[76] Balbuena P., Li W., Ehrich M. (2011). Assessments of tight junction proteins occludin, claudin 5 and scaffold proteins ZO1 and ZO2 in endothelial cells of the rat blood–brain barrier: Cellular responses to neurotoxicants malathion and lead acetate. Neurotoxicology.

[77] Shi L.Z., Zheng W. (2007). Early lead exposure increases the leakage of the blood-cerebrospinal fluid barrier, in vitro. Hum. Exp. Toxicol..

[78] Hossain M.A., Russell J.C., Miknyoczki S., Ruggeri B., Lal B., Laterra J. (2004). Vascular endothelial growth factor mediates vasogenic edema in acute lead encephalopathy. Ann. Neurol..

[79] Song H., Zheng G., Shen X.-F., Liu X.-Q., Luo W.-J., Chen J.-Y. (2014). Reduction of brain barrier tight junctional proteins by lead exposure: Role of activation of nonreceptor tyrosine kinase Src via chaperon GRP78. Toxicol. Sci..

[80] Behl M., Zhang Y., Shi Y., Cheng J., Du Y., Zheng W. (2010). Lead-induced accumulation of β-amyloid in the choroid plexus: Role of low density lipoprotein receptor protein-1 and protein kinase C. Neurotoxicology.

[81] Markovac J., Goldstein G.W. (1988). Lead activates protein kinase C in immature rat brain microvessels. Toxicol. Appl. Pharmacol..

[82] Liu X., Su P., Meng S., Aschner M., Cao Y., Luo W., Zheng G., Liu M. (2017). Role of matrix metalloproteinase-2/9 (MMP2/9) in lead-induced changes in an in vitro blood-brain barrier model. Int. J. Biol. Sci..

[83] Wang Q., Luo W., Zheng W., Liu Y., Xu H., Zheng G., Dai Z., Zhang W., Chen Y., Chen J. (2007). Iron supplement prevents lead-induced disruption of the blood-brain barrier during rat development. Toxicol. Appl. Pharmacol..

[84] Arnal N., Astiz M., de Alaniz M.J., Marra C.A. (2011). Clinical parameters and biomarkers of oxidative stress in agricultural workers who applied copper-based pesticides. Ecotoxicol. Environ. Saf..

[85] Arnal N., de Alaniz M.J., Marra C.A. (2010). Alterations in copper homeostasis and oxidative stress biomarkers in women using the intrauterine device TCu380A. Toxicol. Lett..

[86] Gao Y., Yang W., Che D., Adams S., Yang L. (2020). Advances in the mechanism of high copper diets in restraining pigs growth. J. Anim. Physiol. Anim. Nutr..

[87] Harris E.D. (1992). Copper as a cofactor and regulator of copper, zinc superoxide dismutase. J. Nutr..

[88] Choi B.S., Zheng W. (2009). Copper transport to the brain by the blood-brain barrier and blood-CSF barrier. Brain Res..

[89] Kardos J., Heja L., Simon A., Jablonkai I., Kovacs R., Jemnitz K. (2018). Copper signalling: Causes and consequences. J. Cell Commun. Signal.

[90] Van Bulck M., Sierra-Magro A., Alarcon-Gil J., Perez-Castillo A., Morales-Garcia J.A. (2019). Novel Approaches for the Treatment of Alzheimer’s and Parkinson’s Disease. Int. J. Mol. Sci..

[91] Strozyk D., Launer L.J., Adlard P.A., Cherny R.A., Tsatsanis A., Volitakis I., Blennow K., Petrovitch H., White L.R., Bush A.I. (2009). Zinc and copper modulate Alzheimer Aβ levels in human cerebrospinal fluid. Neurobiol. Aging.

[92] Bandmann O., Weiss K.H., Kaler S.G. (2015). Wilson’s disease and other neurological copper disorders. Lancet Neurol..

[93] Grover S., Gupta P., Kumar A., Mahajan H. (2006). Extensive gray & white matter abnormalities in Wilson’s disease: A case report. Indian J. Radiol. Imaging.

[94] Trickler W.J., Lantz S.M., Schrand A.M., Robinson B.L., Newport G.D., Schlager J.J., Paule M.G., Slikker W., Biris A.S., Hussain S.M. (2012). Effects of copper nanoparticles on rat cerebral microvessel endothelial cells. Nanomedicine.

[95] Quamar S., Kumar J., Mishra A., Flora S. (2019). Oxidative stress and neurobehavioral changes in rats following copper exposure and their response to MiADMSA and d-pencillamine. Toxicol. Res. Appl..

[96] Zhu X., Victor T.W., Ambi A., Sullivan J.K., Hatfield J., Xu F., Miller L.M., Van Nostrand W.E. (2020). Copper accumulation and the effect of chelation treatment on cerebral amyloid angiopathy compared to parenchymal amyloid plaques. Metallomics.

[97] Lamoke F., Mazzone V., Persichini T., Maraschi A., Harris M.B., Venema R.C., Colasanti M., Gliozzi M., Muscoli C., Bartoli M. (2015). Amyloid β peptide-induced inhibition of endothelial nitric oxide production involves oxidative stress-mediated constitutive eNOS/HSP90 interaction and disruption of agonist-mediated Akt activation. J. Neuroinflamm..

[98] Agarwal R., Kushwaha S.S., Tripathi C., Singh N., Chhillar N. (2008). Serum copper in Alzheimer’s disease and vascular dementia. Indian J. Clin. Biochem..

[99] Xiao Y., Yuan Y., Liu Y., Yu Y., Jia N., Zhou L., Wang H., Huang S., Zhang Y., Yang H. (2019). Circulating Multiple Metals and Incident Stroke in Chinese Adults: The Dongfeng-Tongji Cohort. Stroke.

[100] Wang J., Chen J., Tang Z., Li Y., Hu L., Pan J. (2016). The effects of copper on brain microvascular endothelial cells and claudin via apoptosis and oxidative stress. Biol. Trace Elem. Res..

[101] Bar-Or D., Thomas G.W., Yukl R.L., Rael L.T., Shimonkevitz R.P., Curtis C.G., Winkler J.V. (2003). Copper stimulates the synthesis and release of interleukin-8 in human endothelial cells: A possible early role in systemic inflammatory responses. Shock.

[102] Sharma H.S., Hussain S., Schlager J., Ali S.F., Sharma A. (2010). Influence of nanoparticles on blood–brain barrier permeability and brain edema formation in rats. Brain Edema XIV.

[103] Schrag M., Crofton A., Zabel M., Jiffry A., Kirsch D., Dickson A., Mao X.W., Vinters H.V., Domaille D.W., Chang C.J. (2011). Effect of cerebral amyloid angiopathy on brain iron, copper, and zinc in Alzheimer’s disease. J. Alzheimers Dis..

[104] Foidl B.M., Humpel C. (2019). Chronic treatment with five vascular risk factors causes cerebral amyloid angiopathy but no Alzheimer pathology in C57BL6 mice. Brain Behav. Immun..

[105] Kitazawa M., Cheng D., LaFerla F.M. (2009). Chronic copper exposure exacerbates both amyloid and tau pathology and selectively dysregulates cdk5 in a mouse model of AD. J. Neurochem..

[106] Nunes K.Z., Fioresi M., Marques V.B., Vassallo D.V. (2018). Acute copper overload induces vascular dysfunction in aortic rings due to endothelial oxidative stress and increased nitric oxide production. J. Toxicol. Environ. Health.

[107] Seo Y., Cho Y.-S., Huh Y.-D., Park H. (2016). Copper ion from Cu_2_O crystal induces AMPK-mediated autophagy via superoxide in endothelial cells. Mol. Cells.

[108] Wei H., Zhang W.-J., Leboeuf R., Frei B. (2014). Copper induces--and copper chelation by tetrathiomolybdate inhibits--endothelial activation in vitro. Redox. Rep..

[109] Agrawal S., Flora G., Bhatnagar P., Flora S.J.S. (2014). Comparative oxidative stress, metallothionein induction and organ toxicity following chronic exposure to arsenic, lead and mercury in rats. Cell Mol. Biol..

[110] Aschner M., Aschner J.L. (1990). Mercury neurotoxicity: Mechanisms of blood-brain barrier transport. Neurosci. Biobehav. Rev..

[111] Akkoyun H.T. (2018). Effect of boric acid on some elemental levels on rat’s liver and kidney tissues during mercury chloride exposure. Cell Mol. Biol..

[112] Cipollini V., Troili F., Giubilei F. (2019). Emerging Biomarkers in Vascular Cognitive Impairment and Dementia: From Pathophysiological Pathways to Clinical Application. Int. J. Mol. Sci..

[113] Houston M.C. (2011). Role of mercury toxicity in hypertension, cardiovascular disease, and stroke. J. Clin. Hypertens..

[114] Siblerud R., Mutter J., Moore E., Naumann J., Walach H. (2019). A Hypothesis and Evidence That Mercury May be an Etiological Factor in Alzheimer’s Disease. Int. J. Environ. Res. Public Health.

[115] Ware R.A., Louis W.C., Burkholder P.M. (1974). An ultrastructural study on the blood-brain barrier dysfunction following mercury intoxication. Acta Neuropathol..

[116] Usuki F., Yasutake A., Umehara F., Tokunaga H., Matsumoto M., Eto K., Ishiura S., Higuchi I. (2001). In vivo protection of a water-soluble derivative of vitamin E, Trolox, against methylmercury-intoxication in the rat. Neurosci. Lett..

[117] Hirooka T., Fujiwara Y., Yamamoto C., Yasutake A., Kaji T. (2007). Methylmercury retards the repair of wounded monolayer of human brain microvascular endothelial cells by inhibiting their proliferation without nonspecific cell damage. J. Health Sci..

[118] Wierzbicki R., Prażanowski M., Michalska M., Krajewska U., Mielicki W.P. (2002). Disorders in blood coagulation in humans occupationally exposed to mercuric vapors. J. Trace Elem. Exp. Med..

[119] Hirooka T., Fujiwara Y., Inoue S., Shinkai Y., Yamamoto C., Satoh M., Yasutake A., Eto K., Kaji T. (2009). Suppression of fibroblast growth factor-2 expression: Possible mechanism underlying methylmercury-induced inhibition of the repair of wounded monolayers of cultured human brain microvascular endothelial cells. J. Toxicol. Sci..

[120] Ferrara N., Gerber H.-P., LeCouter J. (2003). The biology of VEGF and its receptors. Nat. Med..

[121] Wiggers G.A., Furieri L.B., Briones A.M., Avendaño M.S., Peçanha F.M., Vassallo D.V., Salaices M., Alonso M.J. (2016). Cerebrovascular endothelial dysfunction induced by mercury exposure at low concentrations. Neurotoxicology.

[122] Bjorklund G., Tinkov A.A., Dadar M., Rahman M.M., Chirumbolo S., Skalny A.V., Skalnaya M.G., Haley B.E., Ajsuvakova O.P., Aaseth J. (2019). Insights into the Potential Role of Mercury in Alzheimer’s Disease. J. Mol. Neurosci..

[123] Mutter J., Curth A., Naumann J., Deth R., Walach H. (2010). Does inorganic mercury play a role in Alzheimer’s disease? A systematic review and an integrated molecular mechanism. J. Alzheimers Dis..

[124] Mutter J., Naumann J., Schneider R., Walach H. (2007). Mercury and Alzheimer’s disease. Fortschr. Neurol. Psychiatr..

[125] Kishimoto T., Oguri T., Abe M., Kajitani H., Tada M. (1995). Inhibitory effect of methylmercury on migration and tube formation by cultured human vascular endothelial cells. Arch. Toxicol..

[126] Takahashi T., Fujimura M., Koyama M., Kanazawa M., Usuki F., Nishizawa M., Shimohata T. (2017). Methylmercury causes blood-brain barrier damage in rats via upregulation of vascular endothelial growth factor expression. PLoS ONE.

[127] Chang L.W., Hartmann H.A. (1972). Blood-brain barrier dysfunction in experimental mercury intoxication. Acta Neuropathol..

[128] Yoshida E., Kurita M., Eto K., Kumagai Y., Kaji T. (2017). Methylmercury promotes prostacyclin release from cultured human brain microvascular endothelial cells via induction of cyclooxygenase-2 through activation of the EGFR-p38 MAPK pathway by inhibiting protein tyrosine phosphatase 1B activity. J. Toxicol..

[129] Ghizoni H., de Souza V., Straliotto M.R., de Bem A.F., Farina M., Hort M.A. (2017). Superoxide anion generation and oxidative stress in methylmercury-induced endothelial toxicity in vitro. Toxicol. In Vitro.

[130] Song J.-W., Choi B.-S. (2013). Mercury induced the accumulation of amyloid beta (Aβ) in PC12 cells: The role of production and degradation of Aβ. Toxicol. Res..

[131] Baker B.A., Cassano V.A., Murray C., Exposure A.T.F.o.A. (2018). Arsenic Exposure, Assessment, Toxicity, Diagnosis, and Management: Guidance for Occupational and Environmental Physicians. J. Occup. Environ. Med..

[132] Sen P., Biswas T. (2013). Arsenic: The largest mass poisoning of a population in history. BMJ.

[133] Majid Cheraghali A., Haghqoo S., Shalviri G., Shariati Y.R., Ghassemi M., Khosravi S. (2007). Fatalities following skin exposure to arsenic. Clin. Toxicol..

[134] Rezaei M., Khodayar M.J., Seydi E., Soheila A., Parsi I.K. (2017). Acute, but not Chronic, Exposure to Arsenic Provokes Glucose Intolerance in Rats: Possible Roles for Oxidative Stress and the Adrenergic Pathway. Can. J. Diabetes.

[135] Huang C.F., Chen Y.W., Yang C.Y., Tsai K.S., Yang R.S., Liu S.H. (2011). Arsenic and diabetes: Current perspectives. Kaohsiung J. Med. Sci..

[136] Aposhian H.V., Zakharyan R.A., Avram M.D., Sampayo-Reyes A., Wollenberg M.L. (2004). A review of the enzymology of arsenic metabolism and a new potential role of hydrogen peroxide in the detoxication of the trivalent arsenic species. Toxicol. Appl. Pharmacol..

[137] Douillet C., Huang M.C., Saunders R.J., Dover E.N., Zhang C., Styblo M. (2017). Knockout of arsenic (+3 oxidation state) methyltransferase is associated with adverse metabolic phenotype in mice: The role of sex and arsenic exposure. Arch. Toxicol..

[138] Benramdane L., Accominotti M., Fanton L., Malicier D., Vallon J.J. (1999). Arsenic speciation in human organs following fatal arsenic trioxide poisoning--a case report. Clin. Chem..

[139] Molin Y., Frisk P., Ilback N.G. (2009). Arsenic trioxide affects the trace element balance in tissues in infected and healthy mice differently. Anticancer Res..

[140] Wang C.H., Jeng J.S., Yip P.K., Chen C.L., Hsu L.I., Hsueh Y.M., Chiou H.Y., Wu M.M., Chen C.J. (2002). Biological gradient between long-term arsenic exposure and carotid atherosclerosis. Circulation.

[141] Luo J.H., Qiu Z.Q., Shu W.Q., Zhang Y.Y., Zhang L., Chen J.A. (2009). Effects of arsenic exposure from drinking water on spatial memory, ultra-structures and NMDAR gene expression of hippocampus in rats. Toxicol. Lett..

[142] Chattopadhyay S., Bhaumik S., Nag Chaudhury A., Das Gupta S. (2002). Arsenic induced changes in growth development and apoptosis in neonatal and adult brain cells in vivo and in tissue culture. Toxicol. Lett..

[143] Piao F., Ma N., Hiraku Y., Murata M., Oikawa S., Cheng F., Zhong L., Yamauchi T., Kawanishi S., Yokoyama K. (2005). Oxidative DNA damage in relation to neurotoxicity in the brain of mice exposed to arsenic at environmentally relevant levels. J. Occup. Health.

[144] Meliker J.R., Wahl R.L., Cameron L.L., Nriagu J.O. (2007). Arsenic in drinking water and cerebrovascular disease, diabetes mellitus, and kidney disease in Michigan: A standardized mortality ratio analysis. J. Environ. Health.

[145] Wu M.M., Kuo T.L., Hwang Y.H., Chen C.J. (1989). Dose-response relation between arsenic concentration in well water and mortality from cancers and vascular diseases. Am. J. Epidemiol..

[146] Tsinovoi C.L., Xun P., McClure L.A., Carioni V.M.O., Brockman J.D., Cai J., Guallar E., Cushman M., Unverzagt F.W., Howard V.J. (2018). Arsenic Exposure in Relation to Ischemic Stroke: The Reasons for Geographic and Racial Differences in Stroke Study. Stroke.

[147] Mateen F.J., Grau-Perez M., Pollak J.S., Moon K.A., Howard B.V., Umans J.G., Best L.G., Francesconi K.A., Goessler W., Crainiceanu C. (2017). Chronic arsenic exposure and risk of carotid artery disease: The Strong Heart Study. Environ. Res..

[148] Chen Y., Wu F., Graziano J.H., Parvez F., Liu M., Paul R.R., Shaheen I., Sarwar G., Ahmed A., Islam T. (2013). Arsenic exposure from drinking water, arsenic methylation capacity, and carotid intima-media thickness in Bangladesh. Am. J. Epidemiol..

[149] Balakumar P., Kaur T., Singh M. (2008). Potential target sites to modulate vascular endothelial dysfunction: Current perspectives and future directions. J. Toxicol..

[150] Thompson C.S., Hakim A.M. (2009). Living beyond our physiological means: Small vessel disease of the brain is an expression of a systemic failure in arteriolar function: A unifying hypothesis. Stroke.

[151] Huang J., Li J., Feng C. (2018). Blood-Brain Barrier Damage as the Starting Point of Leukoaraiosis Caused by Cerebral Chronic Hypoperfusion and Its Involved Mechanisms: Effect of Agrin and Aquaporin-4. Biomed. Res. Int..

[152] Pantoni L., Gorelick P.B. (2014). Cerebral Small Vessel Disease.

[153] De Caroli M., Furini A., DalCorso G., Rojas M., Di Sansebastiano G.P. (2020). Endomembrane Reorganization Induced by Heavy Metals. Plants.

[154] Zarazua S., Perez-Severiano F., Delgado J.M., Martinez L.M., Ortiz-Perez D., Jimenez-Capdeville M.E. (2006). Decreased nitric oxide production in the rat brain after chronic arsenic exposure. Neurochem. Res..

[155] Muller S.M., Ebert F., Raber G., Meyer S., Bornhorst J., Huwel S., Galla H.J., Francesconi K.A., Schwerdtle T. (2018). Effects of arsenolipids on in vitro blood-brain barrier model. Arch. Toxicol..

[156] Chen S.C., Chang C.Y., Lin M.L. (2018). Vascular Hyperpermeability Response in Animals Systemically Exposed to Arsenic. Int. J. Med. Sci..

[157] Chen G., Mao J., Zhao J., Zhang Y., Li T., Wang C., Xu L., Hu Q., Wang X., Jiang S. (2016). Arsenic trioxide mediates HAPI microglia inflammatory response and the secretion of inflammatory cytokine IL-6 via Akt/NF-kappaB signaling pathway. Regul. Toxicol. Pharmacol..

[158] Yang J., Wang C., Nie X., Shi S., Xiao J., Ma X., Dong X., Zhang Y., Han J., Li T. (2015). Perfluorooctane sulfonate mediates microglial activation and secretion of TNF-alpha through Ca(2)(+)-dependent PKC-NF-small ka, CyrillicB signaling. Int. Immunopharmacol..

[159] Prakash C., Soni M., Kumar V. (2016). Mitochondrial oxidative stress and dysfunction in arsenic neurotoxicity: A review. J. Appl. Toxicol..

[160] Kharroubi W., Haj Ahmed S., Nury T., Andreoletti P., Sakly R., Hammami M., Lizard G. (2017). Mitochondrial dysfunction, oxidative stress and apoptotic induction in microglial BV-2 cells treated with sodium arsenate. J. Environ. Sci..

[161] Wang X., Meng D., Chang Q., Pan J., Zhang Z., Chen G., Ke Z., Luo J., Shi X. (2010). Arsenic inhibits neurite outgrowth by inhibiting the LKB1-AMPK signaling pathway. Environ. Health Perspect..

[162] Anwar-Mohamed A., Elshenawy O.H., El-Sherbeni A.A., Abdelrady M., El-Kadi A.O. (2014). Acute arsenic treatment alters arachidonic acid and its associated metabolite levels in the brain of C57Bl/6 mice. Can. J. Physiol. Pharmacol..

[163] Escudero-Lourdes C., Uresti-Rivera E.E., Oliva-Gonzalez C., Torres-Ramos M.A., Aguirre-Banuelos P., Gandolfi A.J. (2016). Erratum to: Cortical Astrocytes Acutely Exposed to the Monomethylarsonous Acid (MMA(III)) Show Increased Pro-inflammatory Cytokines Gene Expression that is Consistent with APP and BACE-1 Over-expression. Neurochem. Res..

[164] Ma Y., Ma Z., Yin S., Yan X., Wang J. (2017). Arsenic and fluoride induce apoptosis, inflammation and oxidative stress in cultured human umbilical vein endothelial cells. Chemosphere.

[165] Wang L., Kou M.C., Weng C.Y., Hu L.W., Wang Y.J., Wu M.J. (2012). Arsenic modulates heme oxygenase-1, interleukin-6, and vascular endothelial growth factor expression in endothelial cells: Roles of ROS, NF-kappaB, and MAPK pathways. Arch. Toxicol..

[166] Weng C.Y., Chiou S.Y., Wang L., Kou M.C., Wang Y.J., Wu M.J. (2014). Arsenic trioxide induces unfolded protein response in vascular endothelial cells. Arch. Toxicol..

[167] Chou Y., Tsai C.-H., Ueng K.-C., Tian T.-Y., Chen S.-C., Yeh H.-I. (2007). Endothelial gap junctions are down-regulated by arsenic trioxide. Eur. J. Pharmacol..

[168] Kao Y.H., Yu C.L., Chang L.W., Yu H.S. (2003). Low concentrations of arsenic induce vascular endothelial growth factor and nitric oxide release and stimulate angiogenesis in vitro. Chem. Res. Toxicol..

[169] Manthari R.K., Tikka C., Ommati M.M., Niu R., Sun Z., Wang J., Zhang J., Wang J. (2018). Arsenic induces autophagy in developmental mouse cerebral cortex and hippocampus by inhibiting PI3K/Akt/mTOR signaling pathway: Involvement of blood-brain barrier’s tight junction proteins. Arch. Toxicol..

[170] Sharma B., Sharma P.M. (2013). Arsenic toxicity induced endothelial dysfunction and dementia: Pharmacological interdiction by histone deacetylase and inducible nitric oxide synthase inhibitors. Toxicol. Appl. Pharmacol..

[171] Pi J., Yamauchi H., Sun G., Yoshida T., Aikawa H., Fujimoto W., Iso H., Cui R., Waalkes M.P., Kumagai Y. (2005). Vascular dysfunction in patients with chronic arsenosis can be reversed by reduction of arsenic exposure. Environ. Health Perspect..

[172] Chen J., Kang D., Yan Z., Shen Q., Lou Y., Li Y., Kong A., Pan B., Huang C. (2019). Tissue distribution of tetrabromobisphenol A and cadmium in mixture inhalation exposure. Toxicol. Ind. Health.

[173] Choudhuri S., Liu W.L., Berman N.E., Klaassen C.D. (1996). Cadmium accumulation and metallothionein expression in brain of mice at different stages of development. Toxicol. Lett..

[174] Amuno S., Shekh K., Kodzhahinchev V., Niyogi S. (2020). Neuropathological changes in wild muskrats (Ondatra zibethicus) and red squirrels (Tamiasciurushudsonicus) breeding in arsenic endemic areas of Yellowknife, Northwest Territories (Canada): Arsenic and cadmium accumulation in the brain and biomarkers of oxidative stress. Sci. Total Environ..

[175] Zheng J.L., Yuan S.S., Wu C.W., Lv Z.M. (2016). Acute exposure to waterborne cadmium induced oxidative stress and immunotoxicity in the brain, ovary and liver of zebrafish (Danio rerio). Aquat. Toxicol..

[176] Monaco A., Capriello T., Grimaldi M.C., Schiano V., Ferrandino I. (2017). Neurodegeneration in zebrafish embryos and adults after cadmium exposure. Eur. J. Histochem..

[177] Favorito R., Chiarelli G., Grimaldi M.C., De Bonis S., Lancieri M., Ferrandino I. (2011). Bioaccumulation of cadmium and its cytotoxic effect on zebrafish brain. J. Chem. Ecol..

[178] Xu M.Y., Wang P., Sun Y.J., Yang L., Wu Y.J. (2017). Joint toxicity of chlorpyrifos and cadmium on the oxidative stress and mitochondrial damage in neuronal cells. Food Chem. Toxicol..

[179] Adefegha S.A., Omojokun O.S., Oboh G., Fasakin O., Ogunsuyi O. (2016). Modulatory Effects of Ferulic Acid on Cadmium-Induced Brain Damage. J. Evid. Based Complement. Altern. Med..

[180] Shrivastava A.N., Triller A., Melki R. (2018). Cell biology and dynamics of Neuronal Na+/K+-ATPase in health and diseases. Neuropharmacology.

[181] Zou J., Chen Z., Liang C., Fu Y., Wei X., Lu J., Pan M., Guo Y., Liao X., Xie H. (2018). Trefoil factor 3, cholinesterase and homocysteine: Potential predictors for Parkinson’s disease dementia and vascular parkinsonism dementia in advanced stage. Aging Dis..

[182] Rosenberg G.A. (2016). Matrix metalloproteinase-mediated Neuroinflammation in vascular cognitive impairment of the Binswanger type. Cell. Mol. Neurobiol..

[183] Saleh H.M., El-Sayed Y.S., Naser S.M., Eltahawy A.S., Onoda A., Umezawa M. (2017). Efficacy of alpha-lipoic acid against cadmium toxicity on metal ion and oxidative imbalance, and expression of metallothionein and antioxidant genes in rabbit brain. Environ. Sci. Pollut. Res. Int..

[184] Pulido G., Trevino S., Brambila E., Vazquez-Roque R., Moreno-Rodriguez A., Pena Rosas U., Moran-Perales J.L., Handal Silva A., Guevara J., Flores G. (2019). The Administration of Cadmium for 2, 3 and 4 Months Causes a Loss of Recognition Memory, Promotes Neuronal Hypotrophy and Apoptosis in the Hippocampus of Rats. Neurochem. Res..

[185] Yang X.F., Fan G.Y., Liu D.Y., Zhang H.T., Xu Z.Y., Ge Y.M., Wang Z.L. (2015). Effect of cadmium exposure on the histopathology of cerebral cortex in juvenile mice. Biol. Trace Elem. Res..

[186] Lopez E., Figueroa S., Oset-Gasque M.J., Gonzalez M.P. (2003). Apoptosis and necrosis: Two distinct events induced by cadmium in cortical neurons in culture. Br. J. Pharmacol..

[187] Yan Y., Bian J.C., Zhong L.X., Zhang Y., Sun Y., Liu Z.P. (2012). Oxidative stress and apoptotic changes of rat cerebral cortical neurons exposed to cadmium in vitro. Biomed. Environ. Sci..

[188] Zhang Y.M., Liu X.Z., Lu H., Mei L., Liu Z.P. (2009). Lipid peroxidation and ultrastructural modifications in brain after perinatal exposure to lead and/or cadmium in rat pups. Biomed. Environ. Sci..

[189] Ibiwoye M.O., Matthews Q., Travers K., Foster J.D. (2019). Association of Acute, High-dose Cadmium Exposure with Alterations in Vascular Endothelial Barrier Antigen Expression and Astrocyte Morphology in the Developing Rat Central Nervous System. J. Comp. Pathol..

[190] Wei X., Qi Y., Zhang X., Gu X., Cai H., Yang J., Zhang Y. (2015). ROS act as an upstream signal to mediate cadmium-induced mitophagy in mouse brain. Neurotoxicology.

[191] Valois A.A., Webster W.S. (1987). The choroid plexus and cerebral vasculature as target sites for cadmium following acute exposure in neonatal and adult mice: An autoradiographic and gamma counting study. J. Toxicol..

[192] Wang H., Zhang L., Abel G.M., Storm D.R., Xia Z. (2018). Cadmium exposure impairs cognition and olfactory memory in male C57BL/6 mice. Toxicol. Sci..

[193] Pm M.M., Shahi M.H., Tayyab M., Farheen S., Khanam N., Tabassum S., Ali A. (2019). Cadmium-induced neurodegeneration and activation of noncanonical sonic hedgehog pathway in rat cerebellum. J. Biochem. Mol. Toxicol..

[194] Sies H., Jones D.P. (2020). Reactive oxygen species (ROS) as pleiotropic physiological signalling agents. Nat. Rev. Mol. Cell Biol..

[195] Tafuri S., Cocchia N., Landolfi F., Iorio E.L., Ciani F. (2016). Redoxomics and oxidative stress: From the basic research to the clinical practice. Free Rad. Dis..

[196] Wardlaw J.M., Smith C., Dichgans M. (2013). Mechanisms of sporadic cerebral small vessel disease: Insights from neuroimaging. Lancet Neurol..

[197] Valko M., Morris H., Cronin M.T. (2005). Metals, toxicity and oxidative stress. Curr. Med. Chem..

[198] Shahid M., Pourrut B., Dumat C., Nadeem M., Aslam M., Pinelli E. (2014). Heavy-metal-induced reactive oxygen species: Phytotoxicity and physicochemical changes in plants. Rev. Environ. Contam. Toxicol..

[199] Forstermann U. (2010). Nitric oxide and oxidative stress in vascular disease. Pflugers Arch..

[200] Meza C.A., La Favor J.D., Kim D.-H., Hickner R.C. (2019). Endothelial Dysfunction: Is There a Hyperglycemia-Induced Imbalance of NOX and NOS?. Int. J. Mol. Sci..

[201] Xie H., Ray P.E., Short B.L. (2005). NF-kappaB activation plays a role in superoxide-mediated cerebral endothelial dysfunction after hypoxia/reoxygenation. Stroke.

[202] Wang F., Cao Y., Ma L., Pei H., Rausch W.D., Li H. (2018). Dysfunction of Cerebrovascular Endothelial Cells: Prelude to Vascular Dementia. Front. Aging Neurosci..

[203] Gavard J., Gutkind J.S. (2008). Protein kinase C-related kinase and ROCK are required for thrombin-induced endothelial cell permeability downstream from Galpha12/13 and Galpha11/q. J. Biol. Chem..

[204] Yao L., Romero M.J., Toque H.A., Yang G., Caldwell R.B., Caldwell R.W. (2010). The role of RhoA/Rho kinase pathway in endothelial dysfunction. J. Cardiovasc. Dis. Res..

[205] Romero M.J., Platt D.H., Tawfik H.E., Labazi M., El-Remessy A.B., Bartoli M., Caldwell R.B., Caldwell R.W. (2008). Diabetes-induced coronary vascular dysfunction involves increased arginase activity. Circ. Res..

[206] Brown D.I., Griendling K.K. (2015). Regulation of signal transduction by reactive oxygen species in the cardiovascular system. Circ. Res..

[207] Kawamoto R., Ninomiyax D., Kusunoki T., Kasai Y., Ohtsuka N., Kumagi T. (2016). Oxidative stress is associated with increased arterial stiffness in middle-aged and elderly community-dwelling persons. J. Clin. Gerontol. Geriatr..

[208] Tejovathi B., Suchitra M.M., Suresh V., Reddy V.S., Sachan A., Srinivas Rao P.V., Bitla A.R. (2013). Association of lipid peroxidation with endothelial dysfunction in patients with overt hypothyroidism. Exp. Clin. Endocrinol. Diabetes.

[209] Houston M.C. (2007). The role of mercury and cadmium heavy metals in vascular disease, hypertension, coronary heart disease, and myocardial infarction. Altern. Ther. Health Med..

[210] Tang L., Su J., Liang P. (2017). Modeling cadmium-induced endothelial toxicity using human pluripotent stem cell-derived endothelial cells. Sci. Rep..

[211] Sena C.M., Pereira A.M., Seica R. (2013). Endothelial dysfunction—A major mediator of diabetic vascular disease. Biochim. Biophys. Acta.

[212] Campbell N.R., Lackland D.T., Lisheng L., Niebylski M.L., Nilsson P.M., Zhang X.H. (2015). Using the Global Burden of Disease study to assist development of nation-specific fact sheets to promote prevention and control of hypertension and reduction in dietary salt: A resource from the World Hypertension League. J. Clin. Hypertens..

[213] Chrissobolis S., Miller A.A., Drummond G.R., Kemp-Harper B.K., Sobey C.G. (2011). Oxidative stress and endothelial dysfunction in cerebrovascular disease. Front. Biosci..

[214] Sena C.M., Leandro A., Azul L., Seiça R., Perry G. (2018). Vascular Oxidative Stress: Impact and Therapeutic Approaches. Front. Physiol..

[215] Wu J., Xia S., Kalionis B., Wan W., Sun T. (2014). The role of oxidative stress and inflammation in cardiovascular aging. BioMed. Res. Int..

[216] Laroux F.S. (2004). Mechanisms of inflammation: The good, the bad and the ugly. Front. Biosci..

[217] Liu T., Zhang L., Joo D., Sun S.-C. (2017). NF-κB signaling in inflammation. Signal Transduct. Target Ther..

[218] Blaser H., Dostert C., Mak T.W., Brenner D. (2016). TNF and ROS Crosstalk in Inflammation. Trends Cell Biol..

[219] Barchowsky A., Dudek E.J., Treadwell M.D., Wetterhahn K.E. (1996). Arsenic induces oxidant stress and NF-KB activation in cultured aortic endothelial cells. Free Radic. Biol. Med..

[220] Kaji T., Suzuki M., Yamamoto C., Mishima A., Sakamoto M., Kozuka H. (1995). Severe damage of cultured vascular endothelial cell monolayer after simultaneous exposure to cadmium and lead. Arch. Environ. Contam. Toxicol..

[221] Tsai S.H., Liang Y.C., Chen L., Ho F.M., Hsieh M.S., Lin J.K. (2002). Arsenite stimulates cyclooxygenase-2 expression through activating IkappaB kinase and nuclear factor kappaB in primary and ECV304 endothelial cells. J. Cell. Biochem..

[222] Wei M., Liu J., Xu M., Rui D., Xu S., Feng G., Ding Y., Li S., Guo S. (2016). Divergent Effects of Arsenic on NF-kappaB Signaling in Different Cells or Tissues: A Systematic Review and Meta-Analysis. Int. J. Environ. Res. Public Health.

[223] Zhang H., Park Y., Wu J., Chen X.p., Lee S., Yang J., Dellsperger K.C., Zhang C. (2009). Role of TNF-alpha in vascular dysfunction. Clin. Sci..

[224] Shoamanesh A., Preis S.R., Beiser A.S., Vasan R.S., Benjamin E.J., Kase C.S., Wolf P.A., DeCarli C., Romero J.R., Seshadri S. (2015). Inflammatory biomarkers, cerebral microbleeds, and small vessel disease: Framingham Heart Study. J. Neurol..

[225] Rosenberg G.A. (2009). Inflammation and white matter damage in vascular cognitive impairment. Stroke.

[226] Topakian R., Barrick T.R., Howe F.A., Markus H.S. (2010). Blood-brain barrier permeability is increased in normal-appearing white matter in patients with lacunar stroke and leucoaraiosis. J. Neurol. Neurosurg. Psychiatry.

[227] Candelario-Jalil E., Thompson J., Taheri S., Grossetete M., Adair J.C., Edmonds E., Prestopnik J., Wills J., Rosenberg G.A. (2011). Matrix metalloproteinases are associated with increased blood-brain barrier opening in vascular cognitive impairment. Stroke.

[228] Zhang M., Zhu W., Yun W., Wang Q., Cheng M., Zhang Z., Liu X., Zhou X., Xu G. (2015). Correlation of matrix metalloproteinase-2 single nucleotide polymorphisms with the risk of small vessel disease (SVD). J. Neurol. Sci..

[229] Yi X., Sui G., Zhou Q., Wang C., Lin J., Chai Z., Zhou J. (2019). Variants in matrix metalloproteinase-9 gene are associated with hemorrhagic transformation in acute ischemic stroke patients with atherothrombosis, small artery disease, and cardioembolic stroke. Brain Behav..

[230] Heo J.H., Lucero J., Abumiya T., Koziol J.A., Copeland B.R., del Zoppo G.J. (1999). Matrix metalloproteinases increase very early during experimental focal cerebral ischemia. J. Cereb. Blood Flow Metab..

[231] Yang Y., Estrada E.Y., Thompson J.F., Liu W., Rosenberg G.A. (2007). Matrix metalloproteinase-mediated disruption of tight junction proteins in cerebral vessels is reversed by synthetic matrix metalloproteinase inhibitor in focal ischemia in rat. J. Cereb. Blood Flow Metab..

[232] Chandler S., Coates R., Gearing A., Lury J., Wells G., Bone E. (1995). Matrix metalloproteinases degrade myelin basic protein. Neurosci. Lett..

[233] Candelario-Jalil E., Yang Y., Rosenberg G.A. (2009). Diverse roles of matrix metalloproteinases and tissue inhibitors of metalloproteinases in neuroinflammation and cerebral ischemia. Neuroscience.

[234] Hill J.W., Nemoto E.M. (2015). Matrix-derived inflammatory mediator N-acetyl proline-glycine-proline is neurotoxic and upregulated in brain after ischemic stroke. J. Neuroinflamm..

[235] Gerwien H., Hermann S., Zhang X., Korpos E., Song J., Kopka K., Faust A., Wenning C., Gross C.C., Honold L. (2016). Imaging matrix metalloproteinase activity in multiple sclerosis as a specific marker of leukocyte penetration of the blood-brain barrier. Sci. Transl. Med..

[236] Boroujerdi A., Welser-Alves J.V., Milner R. (2015). Matrix metalloproteinase-9 mediates post-hypoxic vascular pruning of cerebral blood vessels by degrading laminin and claudin-5. Angiogenesis.

[237] Brilha S., Ong C.W.M., Weksler B., Romero N., Couraud P.O., Friedland J.S. (2017). Matrix metalloproteinase-9 activity and a downregulated Hedgehog pathway impair blood-brain barrier function in an in vitro model of CNS tuberculosis. Sci. Rep..

[238] Zhang H.T., Zhang P., Gao Y., Li C.L., Wang H.J., Chen L.C., Feng Y., Li R.Y., Li Y.L., Jiang C.L. (2017). Early VEGF inhibition attenuates blood-brain barrier disruption in ischemic rat brains by regulating the expression of MMPs. Mol. Med. Rep..

[239] Egashira Y., Zhao H., Hua Y., Keep R.F., Xi G. (2015). White Matter Injury After Subarachnoid Hemorrhage: Role of Blood-Brain Barrier Disruption and Matrix Metalloproteinase-9. Stroke.

[240] Petty G.W., Brown R.D., Whisnant J.P., Sicks J.D., O’Fallon W.M., Wiebers D.O. (2000). Ischemic stroke subtypes: A population-based study of functional outcome, survival, and recurrence. Stroke.

[241] Rempe R.G., Hartz A.M.S., Soldner E.L.B., Sokola B.S., Alluri S.R., Abner E.L., Kryscio R.J., Pekcec A., Schlichtiger J., Bauer B. (2018). Matrix Metalloproteinase-Mediated Blood-Brain Barrier Dysfunction in Epilepsy. J. Neurosci..

[242] Yan W., Zhao X., Chen H., Zhong D., Jin J., Qin Q., Zhang H., Ma S., Li G. (2016). beta-Dystroglycan cleavage by matrix metalloproteinase-2/-9 disturbs aquaporin-4 polarization and influences brain edema in acute cerebral ischemia. Neuroscience.

[243] Qiu G.P., Xu J., Zhuo F., Sun S.Q., Liu H., Yang M., Huang J., Lu W.T., Huang S.Q. (2015). Loss of AQP4 polarized localization with loss of beta-dystroglycan immunoreactivity may induce brain edema following intracerebral hemorrhage. Neurosci. Lett..

[244] Zhang X., Gu Y., Li P., Jiang A., Sheng X., Jin X., Shi Y., Li G. (2019). Matrix Metalloproteases-Mediated Cleavage on beta-Dystroglycan May Play a Key Role in the Blood-Brain Barrier After Intracerebral Hemorrhage in Rats. Med. Sci. Monit..

[245] Kato J., Hayashi M.K., Aizu S., Yukutake Y., Takeda J., Yasui M. (2013). A general anaesthetic propofol inhibits aquaporin-4 in the presence of Zn(2)(+). Biochem. J..

[246] Rao K.V., Jayakumar A.R., Reddy P.V., Tong X., Curtis K.M., Norenberg M.D. (2010). Aquaporin-4 in manganese-treated cultured astrocytes. Glia.

[247] Gunnarson E., Axehult G., Baturina G., Zelenin S., Zelenina M., Aperia A. (2005). Lead induces increased water permeability in astrocytes expressing aquaporin 4. Neuroscience.

[248] Vella J., Zammit C., Di Giovanni G., Muscat R., Valentino M. (2015). The central role of aquaporins in the pathophysiology of ischemic stroke. Front. Cell Neurosci..

[249] Zhang X., Zhao H.H., Li D., Li H.P. (2019). Neuroprotective effects of matrix metalloproteinases in cerebral ischemic rats by promoting activation and migration of astrocytes and microglia. Brain. Res. Bull..

[250] Chelluboina B., Klopfenstein J.D., Pinson D.M., Wang D.Z., Vemuganti R., Veeravalli K.K. (2015). Matrix Metalloproteinase-12 Induces Blood-Brain Barrier Damage After Focal Cerebral Ischemia. Stroke.

[251] Lee J.Y., Choi H.Y., Ahn H.J., Ju B.G., Yune T.Y. (2014). Matrix metalloproteinase-3 promotes early blood-spinal cord barrier disruption and hemorrhage and impairs long-term neurological recovery after spinal cord injury. Am. J. Pathol..

[252] Rosenberg G.A. (2009). Matrix metalloproteinases and their multiple roles in neurodegenerative diseases. Lancet Neurol..

[253] Raffetto J.D., Khalil R.A. (2008). Matrix metalloproteinases and their inhibitors in vascular remodeling and vascular disease. Biochem. Pharmacol..

[254] Tan B.L., Norhaizan M.E., Liew W.-P.-P., Sulaiman Rahman H. (2018). Antioxidant and Oxidative Stress: A Mutual Interplay in Age-Related Diseases. Front. Pharmacol..

[255] Flora S. (2007). Role of free radicals and antioxidants in health and disease. Cell. Mol. Biol..

[256] Flora S.J. (2011). Arsenic-induced oxidative stress and its reversibility. Free Radic. Biol. Med..

[257] Flora S.J. (2009). Structural, chemical and biological aspects of antioxidants for strategies against metal and metalloid exposure. Oxid. Med. Cell Longev..

[258] Sharma A., Flora S.J.S. (2018). Nutritional management can assist a significant role in alleviation of arsenicosis. J. Trace Elem. Med. Biol..

[259] Gupta S., Sodhi S., Mahajan V. (2009). Correlation of antioxidants with lipid peroxidation and lipid profile in patients suffering from coronary artery disease. Expert Opin. Ther. Targets.

[260] Jozefczak M., Remans T., Vangronsveld J., Cuypers A. (2012). Glutathione is a key player in metal-induced oxidative stress defenses. Int. J. Mol. Sci..

[261] Rubino F.M. (2015). Toxicity of Glutathione-Binding Metals: A Review of Targets and Mechanisms. Toxics.

[262] Zheng W., Aschner M., Ghersi-Egea J.-F. (2003). Brain barrier systems: A new frontier in metal neurotoxicological research. Toxicol. Appl. Pharmacol..

[263] Shimizu H., Kiyohara Y., Kato I., Kitazono T., Tanizaki Y., Kubo M., Ueno H., Ibayashi S., Fujishima M., Iida M. (2004). Relationship between plasma glutathione levels and cardiovascular disease in a defined population: The Hisayama study. Stroke.

[264] Agrawal S., Bhatnagar P., Flora S.J.S. (2015). Changes in tissue oxidative stress, brain biogenic amines and acetylcholinesterase following co-exposure to lead, arsenic and mercury in rats. Food Chem. Toxicol..

[265] Shukla A., Shukla G.S., Srimal R. (1996). Cadmium-induced alterations in blood-brain barrier permeability and its possible correlation with decreased microvessel antioxidant potential in rat. Hum. Exp. Toxicol..

[266] Chang W.C., Chen S.-H., Wu H.-L., Shi G.-Y., Murota S.-i., Morita I. (1991). Cytoprotective effect of reduced glutathione in arsenical-induced endothelial cell injury. J. Toxicol..

[267] Song J., Park J., Oh Y., Lee J.E. (2015). Glutathione Suppresses Cerebral Infarct Volume and Cell Death after Ischemic Injury: Involvement of FOXO3 Inactivation and Bcl2 Expression. Oxid. Med. Cell. Longev..

[268] Flora S.J.S., Pachauri V. (2010). Chelation in metal intoxication. Int. J. Environ. Res. Public Health.

[269] Bergsland N., Tavazzi E., Schweser F., Jakimovski D., Hagemeier J., Dwyer M.G., Zivadinov R. (2019). Targeting Iron Dyshomeostasis for Treatment of Neurodegenerative Disorders. CNS Drugs.

[270] Baldari S., Di Rocco G., Toietta G. (2020). Current Biomedical Use of Copper Chelation Therapy. Int. J. Mol. Sci..

[271] Flora S.J.S., Bhadauria S., Kannan G., Singh N. (2007). Arsenic induced oxidative stress and the role of antioxidant supplementation during chelation: A review. J. Environ. Biol..

[272] Sidhu M.S., Saour B.M., Boden W.E. (2014). A TACTful reappraisal of chelation therapy in cardiovascular disease. Nat. Rev. Cardiol..

[273] Anderson T.J., Hubacek J., Wyse D.G., Knudtson M.L. (2003). Effect of chelation therapy on endothelial function in patients with coronary artery disease: PATCH substudy. J. Am. Coll. Cardiol..

[274] Seely D.M.R., Wu P., Mills E.J. (2005). EDTA chelation therapy for cardiovascular disease: A systematic review. BMC Cardiovasc Disord..

[275] Lamas G.A., Ergui I. (2016). Chelation therapy to treat atherosclerosis, particularly in diabetes: Is it time to reconsider?. Expert Rev. Cardiovasc. Ther..

[276] Solenkova N.V., Newman J.D., Berger J.S., Thurston G., Hochman J.S., Lamas G.A. (2014). Metal pollutants and cardiovascular disease: Mechanisms and consequences of exposure. Am. Heart J..

[277] Sompamit K., Kukongviriyapan U., Donpunha W., Nakmareong S., Kukongviriyapan V. (2010). Reversal of cadmium-induced vascular dysfunction and oxidative stress by meso-2,3-dimercaptosuccinic acid in mice. Toxicol. Lett..

[278] Flora S.J.S. (2020). Preventive and Therapeutic Strategies for Acute and Chronic Human Arsenic Exposure. Arsenic in Drinking Water and Food.

[279] Satarug S., Moore M.R. (2004). Adverse health effects of chronic exposure to low-level cadmium in foodstuffs and cigarette smoke. Environ. Health Persp..

[280] Blanchemanche S., Marette S., Roosen J., Verger P. (2010). ‘Do not eat fish more than twice a week’. Rational choice regulation and risk communication: Uncertainty transfer from risk assessment to public. Health Risk Soc..

[281] Remor A.P., Totti C.C., Moreira D.A., Dutra G.P., Heuser V.D., Boeira J.M. (2009). Occupational exposure of farm workers to pesticides: Biochemical parameters and evaluation of genotoxicity. Environ. Internat..

